# Agreement Errors Disrupt Eye Movements in Reading Only When They Are Detected

**DOI:** 10.1162/OPMI.a.374

**Published:** 2026-07-17

**Authors:** Adrian Staub, Nathan Wolf, Brian Dillon

**Affiliations:** Department of Psychological and Brain Sciences, University of Massachusetts Amherst, Amherst, MA, USA; Department of Linguistics, University of Massachusetts Amherst, Amherst, MA, USA

**Keywords:** reading, eye movements, agreement

## Abstract

Sentence processing models posit that syntactic processing difficulty contributes to the processing time of each word (e.g., Lewis & Vasishth, 2005). On the other hand, the E-Z Reader 10 model of eye movements in reading (Reichle et al., 2009) proposes that syntactic processing affects eye movements only when integration of an input word *fails*. We present two eye movement experiments designed to address this tension. Readers were presented with ungrammatical sentences with a singular subject and a plural verb, but with a plural local noun that often induces an illusion of grammaticality, e.g., *The editor of the magazines are reviewing the articles before publication. After each critical sentence was removed from the screen, the reader made a binary acceptability judgment. When readers rejected the ungrammatical critical sentences, there was also pronounced disruption to incremental reading. However, on the majority of trials readers accepted these sentences, and on those trials the eye movement record revealed only a very small increase in reading time compared to corresponding grammatical sentences; critically, this small effect was entirely explained by the difference between conditions in the lexical surprisal of the verb. Thus, as predicted by E-Z Reader 10, an effect of agreement computation above and beyond the effect of surprisal was in evidence only when agreement computation failed, i.e., the sentence was rejected. We discuss implications for models of incremental sentence processing and reading.

## INTRODUCTION

A major project in psycholinguistics is understanding how linguistic computations are reflected in measurements of incremental processing difficulty, such as the duration and location of eye fixations on a text. Even though most psycholinguistic theory aims to characterize underlying linguistic processes, an understanding of the mapping between the processing operations posited by a psycholinguistic theory and observable behavioral measures is essential for interpretation of experimental results, and is also of intrinsic theoretical interest. It is a widely held assumption that gradient differences in the difficulty of hypothesized linguistic computations, such as retrieval of dependents from memory or the resolution of syntactic ambiguity, produce corresponding differences in incremental processing measures.

In this study, we scrutinize this assumption and ask: Does the difficulty of syntactic processing *generally* affect eye movements during reading, or does syntactic processing impact eye movements only when the reader fails, at least initially, to construct a grammatical parse of a sentence? In two experiments investigating processing of subject-verb agreement, we find clear support for the latter position. On the basis of these results, we argue for revisiting the common assumption that syntactic processing difficulty has a continuous, graded effect on reading time. The largely discrete nature of reading time effects induced by syntactic processing, in our study, offers an important clue to the underlying cognitive processes involved in constructing sentence-level representations of linguistic input. In particular, it highlights error detection (and, perhaps, correction) as a critical component of language comprehension.

### Eye Movements in Reading

The eyes’ forward progress in reading is affected by a range of linguistic factors. A word’s frequency (e.g., Rayner & Duffy, 1986; Staub et al., 2010) and predictability (e.g., Ehrlich & Rayner, 1981; Staub, 2011) influence the duration of eye fixations, and syntactic factors influence both the duration of the eyes’ inspection of a word or phrase and the probability of a regressive eye movement to an earlier location in the text (e.g., Frazier & Rayner, 1982; see Clifton & Staub, 2011, for review). Perhaps the best known demonstrations of such syntactic effects come from experiments with garden path sentences, in which the reader encounters input that disambiguates a syntactically ambiguous sentence toward an analysis that was initially dispreferred; in this case, regressive eye movements to an earlier sentence region are common (e.g., Frazier & Rayner, 1982; von der Malsburg & Vasishth, 2011). Many other varieties of syntactic effects have been investigated, including effects attributable to complexity or memory demands rather than syntactic disambiguation (Bartek et al., 2011; Demberg & Keller, 2008), as well as effects of outright ungrammaticality (Pearlmutter et al., 1999; Sturt, 2003).

Early computational models of eye movements in reading such as SWIFT (e.g., Engbert et al., 2005) and E-Z Reader (Reichle et al., 2003) accounted for the effects of perceptual and lexical factors on fixation durations and locations. We focus here on the E-Z Reader model, in which effects of both lexical frequency and predictability arise due to a mechanism in which a saccade is programmed to leave a currently fixated word when a critical stage of lexical processing (termed *L1*) is complete, with frequency and predictability directly modulating the duration of the L1 stage. As frequency and predictability each increase, the duration of the L1 stage decreases monotonically. (In the earliest versions of E-Z Reader, L1 was identified as a ‘familiarity check’, but in later versions even this informal description was dropped, as the L1 stage was identified only in terms of its eye movement consequences.)

Notably, neither early versions of the E-Z Reader model nor any other contemporaneous model attempted to account for syntactic effects, or other ‘post-lexical’ effects such as the effect of encountering an implausible word (e.g., Rayner et al., 2004). The first eye movement model to explicitly address effects of syntactic processing, or other post-lexical processing, was an updated version of E-Z Reader known as E-Z Reader 10 (Reichle et al., 2009). E-Z Reader 10 introduced a post-lexical integration stage (*I*), whose duration reflects the time required for integration of a word into its syntactic and semantic context. However, the model posits that the duration of the *I* stage usually has no influence on eye movements. Reichle et al. suggested that “a postlexical stage of language processing can run invisibly in the background of ongoing lexical processing” (p. 12); the postlexical processing that is typically required has “no discernable effects on the progression of the eyes through the text” (p. 16). Postlexical processing is assumed to influence eye movements only on those occasions when an input word is *not* successfully attached into the developing syntactic or semantic representation of the sentence; the model terms this *integration failure*. Though E-Z Reader 10 is agnostic as to what underlying psycholinguistic processes result in integration failure, it proposed two distinct varieties of failure: Either postlexical integration lags very far behind ongoing lexical processing (specifically, the *I* stage for word *n* is still not complete once word *n* + 1 has been identified), triggering an error signal, or the *I* stage fails outright; separate model parameters govern the duration of *I* and the probability of outright failure. Either kind of failure is likely to result in a regression to an earlier text region.

E-Z Reader 10’s failure-based mechanism implies that post-lexical processing effects, unlike effects of word frequency and predictability, are not ubiquitous and graded, but are either present or absent: If post-lexical integration of a word into its syntactic or semantic context fails, the forward progress of the eyes is interrupted, but when integration succeeds, difficulty has no overt cost. Thus, input that is somewhat difficult to integrate, but is ultimately integrated in a relatively timely manner, should have no effect on eye movements.

The all-or-none nature of syntactic influence on eye movements in E-Z Reader 10 is notable in light of the fact that many sentence processing models with psycholinguistic origins posit that a word’s processing time is an essentially continuous function of syntactic processing difficulty. For example, in the highly influential activation-based model of Lewis and Vasishth (2005), the processing time of a word is a function of (among other things) the time required to retrieve from working memory the elements that are related to the currently processed word by means of a syntactic dependency (but see Nicenboim & Vasishth, 2018). To take another example, Gibson’s Dependency Locality Theory (Gibson, 1998, 2001) also proposed that there is a syntactic contribution to each word’s processing time, determined by the number of ‘discourse units’ intervening between that word and the previous word to which it is related by a syntactic dependency (for broad coverage evaluation of similar ideas using reading data, see Demberg & Keller, 2008; Isono, 2024).

One intuitive way of deriving reading time predictions from these models is to assume a simple correspondence between reading time and processing time: More processing time means more reading time. This assumption is, we would argue, implicit in many psycholinguistic studies, and is explicit in the famous ‘eye-mind’ hypothesis of Just and Carpenter (1980), which states that, “the eye remains fixated on a word as long as the word is being processed” (p. 330). But this intuitive linking theory is clearly in tension with the all-or-none nature of syntactic processing effects in E-Z Reader 10, where syntactic processing of an input word often makes no contribution to reading time. Engelmann et al. (2013) recognized this tension, and proposed a model of eye movement control wherein continuous memory retrieval times are linked to reading time via a probabilistic and discrete ‘failure’ or ‘error’ signal like one of E-Z Reader 10’s failure mechanisms: When the memory retrievals required to attach a given word are not complete by the time the next word has been identified, a regression to the previous word is initiated. It is worth noting that Engelmann et al. see failures arising in this way as quite common, and they explicitly distinguish these events from the rarer and potentially more catastrophic failures that might arise in an ungrammatical sentence, or upon disambiguation of a garden path sentence, which are not modeled by Engelmann et al. (see Nicenboim & Vasishth, 2018, for a related discussion). What we wish to emphasize in the present context, however, is that in the sentence processing literature as a whole it is common to assume that syntactic processing difficulty generally makes some contribution to word-by-word reading time, rather than only when syntactic attachment (or ‘integration’) fails, or an error is otherwise registered.

### The Intermittent Judgment Paradigm

Previous results using what we will term the *Intermittent Judgment Paradigm* (IJP) provide background for the present study (Huang & Staub, 2021; Staub et al., 2019, 2024). In this paradigm, readers’ eye movements are tracked as they read individual sentences, and after each sentence is removed from the screen, the subject responds by button press to a two-alternative question about that sentence. In a large number of filler trials—larger than the number of experimental trials—these are comprehension questions about the preceding sentence. After the experimental trials, however, the subject is asked, “Was there anything wrong with that sentence?” with the response options *yes* and *no*. Subjects do not know in advance whether a sentence will be followed by a comprehension question or a judgment question. A critical difference from standard linguistic judgment tasks, therefore, is that the subject can be presumed to read the stimulus in a fairly (if not completely) natural manner, as judging the acceptability of sentences is not presented as the main task. A critical difference from the standard eye tracking paradigm, on the other hand, is that for the critical trials it is possible to analyze eye movements contingent on the subject’s acceptance (a *no* response) or rejection (a *yes* response) of the sentence.

Using the IJP, readers have been presented with ungrammatical sentences in which a word is repeated or omitted (Staub et al., 2019, 2024), or two words are transposed (Huang & Staub, 2021). Unsurprisingly, on the trials on which these errors are consciously noticed, eye movement disruption is clearly evident. But readers very often fail to detect the critical errors, and the trials on which readers accept the sentence do not show any sign of eye movement disruption, compared to reading of grammatical control sentences. Staub et al. (2024) argued that this result implicates a form of perceptual (as opposed to post-perceptual) inference in readers’ failure to notice these errors; on many trials, readers’ grammatical knowledge is used to ‘correct’ these errors so rapidly and so unconsciously that the forward movement of the eyes is completely unperturbed.

We may regard a reader’s rejection of an ungrammatical sentence with a repetition, omission, or transposition as reflecting, in E-Z Reader’s terms, integration failure. Of course, readers may sometimes reject a sentence for reasons unrelated to the specific syntactic error that we have inserted; indeed, we know this to be the case, as the false alarm rate in this paradigm— the rate at which readers reject grammatical control sentences—is not zero. However, we assume that when one of the inserted grammatical errors has resulted in a failure to construct a licensed grammatical structure, i.e., integration failure, the reader will reject the sentence. Thus, we assume that when the reader accepts an ungrammatical sentence, integration failure has not occurred^1^. Based on this assumption, the results of these previous studies using the IJP appear to support E-Z Reader 10’s assumption that post-lexical processing difficulty does not affect eye movements unless a discrete failure or error signal is registered. When the ungrammatical lexical material is ‘successfully’ integrated (with the scare quotes meant to indicate that this is a success only in the sense that the integration process results in a grammatical, though possibly non-veridical, representation), the process of integration does not leave a trace in the eye movement record.

### Agreement Processing and Agreement Attraction

Here we deploy the IJP to further investigate the impact of syntactic processing on eye movements. We focus on processing of errors of subject-verb agreement. Previous research has established that when agreement errors occur in certain configurations, they are often not consciously noticed. However, we also know that these agreement errors do show effects in the eye movement record. In the present study, we ask whether a subject-verb agreement error actually impacts eye movements only on trials on which the error is noticed. If so, this would suggest, consistent with the assumptions of E-Z Reader 10, that processing difficulty induced by this syntactic error does not impede the forward movement of the eyes when integration is ultimately ‘successful’.

Consider sentence (1):

(1) *The editor of the magazines are reviewing the articles before publication.

This sentence contains an agreement error: The subject *editor* is singular, while the verb *are* is plural. But in judgment tasks, subjects tend to accept a sentence like (1) much more often than a sentence like (2), which is also ungrammatical, and differs from (1) only in that the non-subject noun *magazine* is singular:

(2) *The editor of the magazine are reviewing the articles before publication.

For example, in three experiments reported by Hammerly et al. (2019), subjects judged sentences like (1) to be unacceptable between 60% and 72% of the time, while judging sentences like (2) to be unacceptable between 80% and 88% of the time; we refer the interested reader to Table 1 of Hammerly et al. (2019) for an extensive list of studies that have presented acceptability judgment data with these (or similar) constructions. The increased acceptability of (1) relative to (2) is referred to as an ‘illusion of grammaticality.’ Note that the illusion is only partial; sentences like (1) are still rejected at a much higher rate than a grammatical control such as (3). The effect of a local noun on agreement computation in both production and comprehension is known as *agreement attraction*.

(3) The editor of the magazine is reviewing the articles before publication.

**Figure 1.**
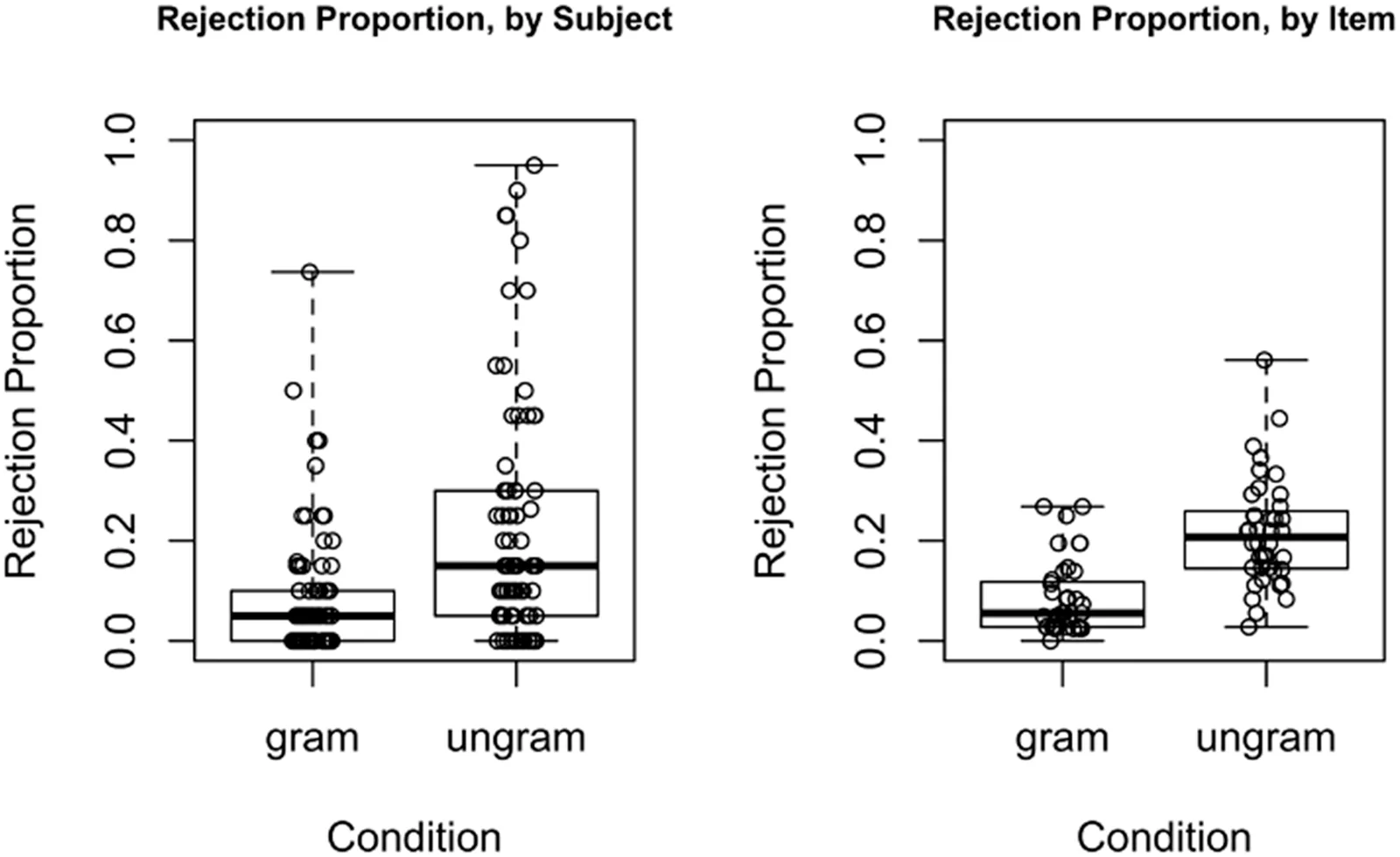
Proportion of sentences rejected in each condition in Experiment 1, by subject (left) and by item (right).

Sentences like (1) have been investigated not only using judgment tasks, but also in incremental comprehension paradigms such as self-paced reading (e.g., Pearlmutter et al., 1999; Wagers et al., 2009) and eyetracking during reading (Dillon et al., 2013; Parker & Phillips, 2017; Pearlmutter et al., 1999). Reading is, on average, less disrupted in a sentence like (1) than in a sentence like (2). Thus, not only does the presence of a plural local noun increase the probability that a reader will incorrectly judge a sentence with a singular subject and plural verb to be grammatical, but the plural local noun also reduces the disruption caused by the agreement violation in the course of incremental processing. But again, this reduction is only partial; reading is still somewhat disrupted, on average, in a sentence like (1) compared to a grammatical control such as (3); in the reading time studies referenced above that tested all of the relevant conditions (Dillon et al., 2013; Pearlmutter et al., 1999; Wagers et al., 2009), reading times for attraction sentences like (1) fell between those of reading times for ungrammatical sentences without an attractor, like (2), and reading times for grammatical sentences, like (3); Figures 1 and 3 of Pearlmutter et al. (1999) provide particularly clear examples of this pattern.

There is substantial debate about why sentence (1) is accepted more often than, and is read faster than, sentence (2). The present study does not attempt to arbitrate between accounts of agreement attraction, but it is nevertheless useful to lay out the theoretical alternatives; we return to these accounts in the General Discussion. According to a *feature distortion* account, the plural noun *magazines* in (1) influences the encoding of the subject phrase’s number. Two distinct variants of the feature distortion account are an older *percolation* account (Bock & Eberhard, 1993; Franck et al., 2002), and a version that we refer to as *continuous number valuation*, originally embodied in the Marking and Morphing model (Eberhard et al., 2005), and developed further by Hammerly et al. (2019) and Staub (2009). On a continuous number valuation account, the plural feature on *magazines* influences a continuously valued number feature associated with the subject phrase as a whole; the entire subject phrase *the editor of the magazines* is encoded as being not entirely singular or entirely plural, but something in between. Thus, when the verb *are* is encountered in (1), there is a *partial* match between the number marking of the verb and the subject phrase, rather than a complete mismatch as in (2). Hammerly et al. (2019) illustrated how this representational assumption, combined with an evidence accumulation model of the acceptability judgment process, predicts that (1) should elicit both more variable judgments of acceptability and longer decision latencies than (2), consistent with the data that Hammerly et al. obtained in a series of judgment experiments.

According to a theory grounded in *cue-based retrieval* (e.g., Wagers et al., 2009), on the other hand, the local noun *magazines* exerts its influence not by means of feature distortion, but because of a process of memory retrieval that searches for the agreement controller once the verb has been encountered. In (1) and (2), the verb *are* triggers a search for a noun with the features +*subject* and +*plural*. In neither case is there a noun that matches both cues. In (2), *editor* matches the +*subject* cue, but there is no noun that matches the +*plural* cue. In (1), however, each of the preceding nouns matches one cue; the two nouns are fairly evenly matched as potential agreement controllers, and the local noun, rather than the true subject, is retrieved a substantial proportion of the time.

### The Present Study

The present study uses the IJP to investigate in detail the eye movement disruption that occurs in reading (1) compared to a grammatical control. Unlike most of the previous agreement literature, which has deployed grammatical controls like (3), where the subject, local noun, and verb are all singular, we use grammatical sentences like (4), where each element is plural.

(4) The editors of the magazines are reviewing the articles before publication.

We use (4) rather than (3) as the grammatical control for (1) so that the critical verbal material is identical between the ungrammatical and grammatical sentences.

The previous literature licenses a confident prediction that on average—aggregating across subjects and trials—there will be eye movement disruption in (1) compared to (4). Our main goal is to compare two models of how such eye movement disruption may be distributed across trials. We expect that the disruption associated with the subject-verb agreement mismatch in (1) may be greatest on the trials on which the reader fails to license agreement between the subject and the verb, and therefore concludes, correctly, that the sentence is ungrammatical. However, it may be expected that the agreement mismatch also creates some degree of disruption even when the plural local noun ultimately induces an illusion of grammaticality; the reader may reach the conclusion that agreement is licensed in a sentence such as (1) only after engaging in a relatively difficult process of agreement computation that interrupts, at least briefly, the forward movement of the eyes. But alternatively, disruption may be *entirely* restricted to those occasions on which the reader concludes that the sentence is ungrammatical; there may be no disruption at all in cases in which (1) is accepted. This second possibility embodies E-Z Reader 10’s assumption that syntactic processing difficulty has an effect on eye movements only in cases of integration failure.

We present two eye movement experiments using the IJP. In both experiments, participants read the same 40 critical items in which a complex subject phrase was followed by a plural verb. Each had only two conditions, the grammatical and ungrammatical conditions represented by (4) and (1) above, repeated here as (5a–b):

(5) a. The editors of the magazines are reviewing the articles before publication.
    b. *The editor of the magazines are reviewing the articles before publication.

Each subject read twenty grammatical sentences like (5a), in which both the subject and the local noun are plural, and twenty ungrammatical ones like (5b), in which the subject is singular but the local noun is plural. After these sentences, they were asked to respond to the judgment question, “Was there anything wrong with that sentence?” The two experiments also included the same 100 filler sentences followed by comprehension questions. The experiments differed only in terms of the other judgment items that were also included, as we describe below.

Our statistical analysis of the eye movement data makes two comparisons. One is to compare eye movements on trials when the reader rejects an ungrammatical sentence to trials when the reader accepts an ungrammatical sentence. The other is to compare eye movements on trials when the reader accepts an ungrammatical sentence to trials on which the reader accepts a corresponding grammatical sentence. It is the latter comparison that addresses our critical theoretical question: Does the ungrammaticality in (5b) result in eye movement disruption even when there is no ‘integration failure,’ as indicated by conscious detection of the sentence’s ungrammaticality?

In evaluating this question statistically, we address head-on an important complication related to assessing whether agreement processing *per se* results in any difficulty in (5b) compared to (5a). The word *are* after the left context in (5a) is likely to be more *predictable* (i.e., have lower surprisal; Levy, 2008) than the word *are* after the left context in (5b); the large language model GPT-2 (Radford et al., 2019) estimates the probability of *are* to be .046 and .003 in (5a) and (5b), respectively. Thus, the verb may elicit longer reading times on the basis of surprisal alone, even if agreement computation itself causes no difficulty in (5b) (Arehalli & Linzen, 2020, 2024). The critical question in the present context is whether, on the trials on which there is no conscious detection of ungrammaticality in sentences like (5b), there is evidence of processing slowdown or disruption in the comparison with (5a) that is more pronounced than would be expected entirely on the basis of differences in lexical surprisal.

## EXPERIMENT 1

### Methods

#### Participants.

The research presented here was approved by the University of Massachusetts Institutional Review Board (Protocol #1151). Students at the University of Masachusetts Amherst received course credit for their participation. All reported being speakers of English as a first language, and all reported no history of reading or language disorder. Eighty-one subjects completed the experiment, of whom three were excluded due to technical problems with their data files, and one was excluded due to poor performance on the comprehension questions following filler trials (below 70%), leaving final *n* = 77 (51 female, 25 male, 1 nonbinary, age range from 18 to 23 years, median = 19).

#### Materials.

An initial set of critical items was generated with a prompt to ChatGPT (OpenAI, 2024) to generate sentences that begin with a subject modified by a prepositional phrase followed by a form of the verb *be*. In order to create structural variety in the critical items, we also asked ChatGPT to generate another set of sentences in which the same sentence type was preceded by additional material such as an adverbial phrase.^2^ Sentences generated by ChatGPT were selected, and modified as needed, to create a total of 20 items in which the matrix subject phrase began the sentence (as in 5a–b), and another 20 in which it did not, as in (6a–b):

(6) a. After several delays, the managers of the projects were eventually called in for a meeting.
    b. *After several delays, the manager of the projects were eventually called in for a meeting.

Thirty-five of the forty items were in the present tense, with the remaining five in the past tense. The length of the material after the critical verb varied across items, but all included at least two words after this verb.

One hundred filler sentences, with a wide range of structures and on a wide range of topics, were selected from the materials in Staub (2024); these sentences were originally taken from the Corpus of Contemporary American English (COCA; Davies, 2009). An example is in (7).

(7) Either way I think he’s a prime candidate for a breakout season next year.

A two-alternative comprehension question was included for each filler sentence; in the case of (7) the question asked, “When will the breakout be?,” followed by the alternatives “next year” and “this year”.

Finally, another 40 items from a separate experiment were also followed by the judgment question, “Was there anything wrong with that sentence?” These items occurred in two conditions, one with a prepositional dative structure, and one in which the word *to* was omitted, resulting in a grammatical but implausible double object sentence. An example is in (8):

(8) a. I didn’t realize that Tom tossed the ball to his dog until it was too late and it hit the window.
    b. I didn’t realize that Tom tossed the ball his dog until it was too late and it hit the window.

The critical judgment sentences in each subexperiment were divided into two lists, so that each subject read 20 sentences from each subexperiment in which the correct answer to the judgment question was ‘yes,’ and 20 in which the correct answer was ‘no.’ In sum, each participant read 180 sentences, with 100 followed by a comprehension question, and 80 followed by a judgment question. The 180 sentences were presented in an individually randomized order to each subject, after four practice trials with comprehension questions.

#### Procedure.

Movements of the right eye were recorded, sampling at 1,000 Hz, using an EyeLink 1000 (SR Research, Toronto, ON, Canada) eyetracker, interfaced with a PC computer. Sentences were displayed on a CRT monitor 55 cm from subjects, in 10-point Monaco font. Each sentence was displayed on a single line of text. The resolution of the eyetracker was less than one character. The experiment was implemented with EyeTrack software, and initial stages of data analysis were carried out with Robodoc and EyeDry (https://websites.umass.edu/eyelab/software/).

Subjects were instructed to read for comprehension, and were told that each sentence would be followed by a question; they were not explicitly instructed that there would be two distinct types of questions. A three-point calibration procedure was performed at the start of the experiment and as needed between trials. Each sentence appeared on a single line when the subject fixated a box at the left edge of the monitor. Subjects were instructed to press a button on a hand-held controller when finished reading each sentence, and to press the left or right trigger on the controller to select the appropriate response option as the answer to the question that appeared after the sentence. For the judgment question, NO appeared on the left and YES on the right. Each session lasted approximately 45–50 minutes.

### Results

As noted above, one subject was excluded based on poor performance on the filler comprehension questions. The remaining subjects all achieved at least 76% correct, with a mean of 92.1%. In total, 6 out of 3080 critical trials were lost due to technical errors, leaving 3074 critical trials available for analysis.

We begin with assessment of judgment accuracy. Figure 1 shows the proportion of trials that were rejected (i.e., the subject responded ‘yes’ to the judgment question) in each condition, by subject and by item. There were few rejections of the grammatical sentences (8.5%). However, subjects also rejected the ungrammatical sentences relatively infrequently (21.7%); the median subject rejected only 15% (3 out of 20) of the ungrammatical sentences, while rejecting 5% (1 out of 20) of the grammatical sentences.

**Figure 2.**
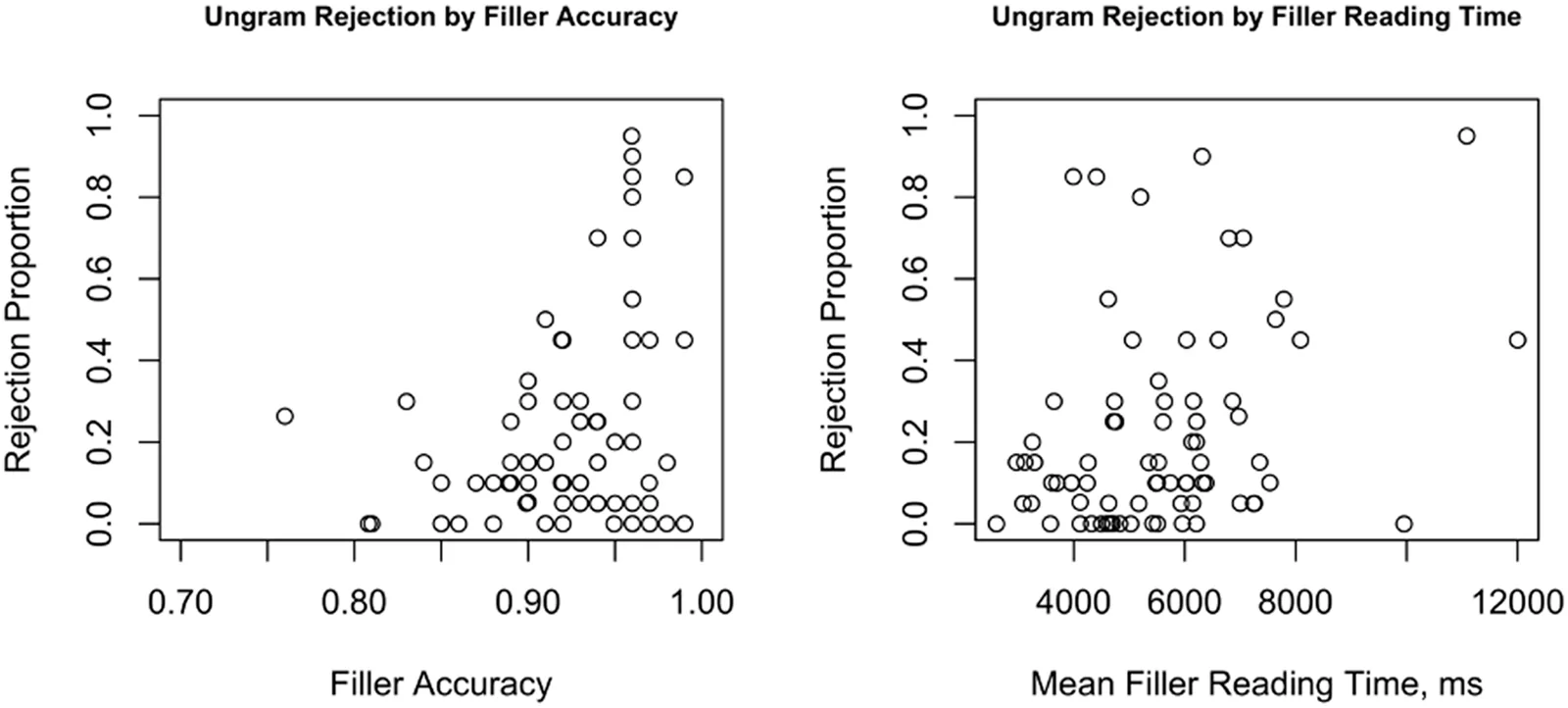
Experiment 1 relationship between filler comprehension accuracy and error detection rate in the critical sentences (left) and between filler reading time and error detection rate in the critical sentences (right).

Previous experiments using the IJP (Huang & Staub, 2021; Staub et al., 2019, 2024) found that subjects’ reading time on the filler sentences and accuracy on the filler questions is modestly correlated with detection of errors on critical judgment trials; slower readers are somewhat better at detecting errors, and readers who perform better on the comprehension questions are also somewhat better at detecting errors. Those patterns are present here as well, as shown in Figure 2. Filler comprehension accuracy and filler reading time happen to demonstrate essentially identical correlation with the error detection rate in the critical sentences (*r* = .32, *p* < .01). It is notable that filler accuracy and reading time are themselves only modestly correlated (*r* = .20, *p* = .09).

The mean rejection rate of the implausible double-object sentences (e.g., 8b) in the set of judgment trials associated with the other subexperiment was 70.3%; the median subject rejected 80%, or 16 out of 20. Thus, subjects were quite good at detecting the implausibility in sentences like (8b), while usually failing to detect the agreement violations in sentences like (5b) and (6b). Due to the very low rate of detection of the agreement errors, we wanted to ensure that our participants would indeed recognize agreement errors of this kind when making an untimed, explicit judgment. For approximately the last third of the period of running the experiment, we administered a very brief post-experiment paper and pencil questionnaire. Participants were provided with sentence (5b), and asked to judge if the sentence is grammatical. If they circled ‘no, ’ they were then asked to write an explanation. Of the 28 participants who received this questionnaire, 20 (71.4%) stated that the sentence is ungrammatical, and these 20 all provided an explanation in terms of subject-verb agreement. Thus, subjects do detect these agreement violations at a much higher rate when grammaticality judgment is the explicit task.

For the purpose of analysis of the eye movement data, we divided each sentence into four regions, as shown in (9):

(9) a. The editors of the\ magazines\ are reviewing\ the articles before publication.
    b. *The editor of the\ magazines\ are reviewing\ the articles before publication.

We report analysis of eye movement measures for the second, third, and fourth regions of the sentence, which were identical across the two conditions. We refer to these as the pre-target, target, and final regions, respectively. The pre-target region consisted of the plural local noun. The target region consisted of the critical plural form of the verb *to be*, and the following word. We included the following word because the critical verb, due to its length, would have been skipped by the eyes rather than directly fixated on many trials. The final region consisted of the rest of the sentence, which was sometimes as short as a single word, but was usually longer, as in (9).

Any trial in which a region was not fixated on first pass reading was excluded from the analysis of that region. The pre-target region was skipped on 20.2% of trials in the grammatical condition, and 17.2% of trials in the ungrammatical condition. The target region and the final region were rarely skipped, as expected for these multi-word regions. The target region was skipped on 4.6% of trials in both conditions. The final region was skipped on 3.3% of trials in the grammatical condition, and 3.8% of trials in the ungrammatical condition.

We analyzed four eye movement measures, which together capture both first-pass processing and later processing of each region. Individual eye fixations less than 80 ms in duration and within one character of a previous or subsequent fixation were incorporated into this neighboring fixation, prior to computing these reading measures. The four measures were: 1) *first pass time*, which is the sum of all eye fixation durations on the region on the reader’s first pass, i.e., before leaving the region to the left or right; 2) *go-past time*, which is the sum of all eye fixation durations starting on the reader’s first fixation on the region, and ending when the reader has moved past the region to the right; this measure includes first pass time, but also includes time spent re-reading both earlier material and the region itself on those trials on which first pass reading terminated with a regressive saccade; 3) *total time*, which is the sum of all fixation durations on the region, including fixations occurring after a regressive saccade from later in the sentence; and 4) the probability that first pass reading of a region terminates with a regression to an earlier sentence region rather than a forward saccade. We also analyzed total sentence reading time, i.e., the time from when the sentence was displayed on the screen to the time the participant pressed a button to remove it.

**Figure 3.**
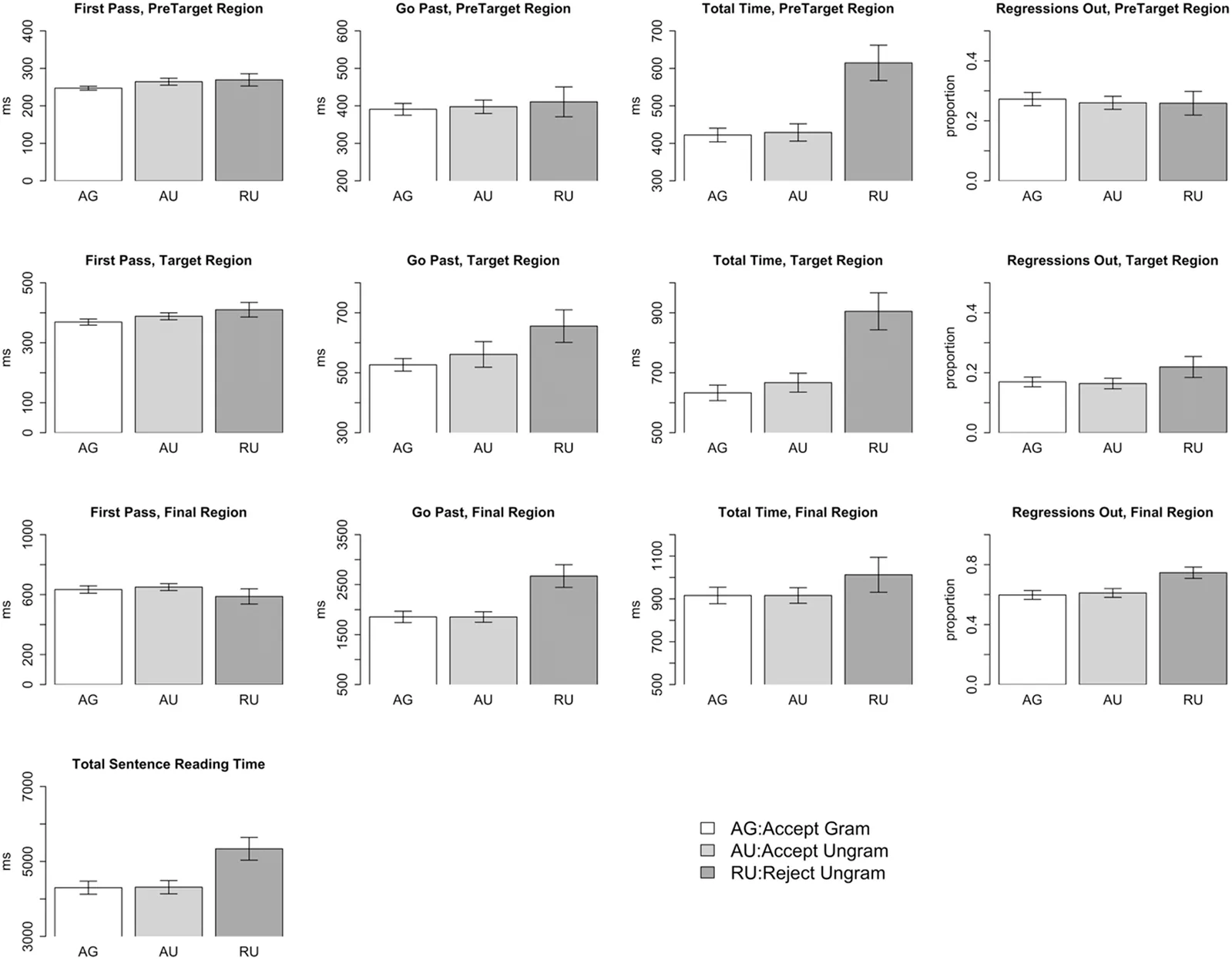
Experiment 1 mean and standard error, by subject, for each eye movement measure on each region, and for total sentence reading time, for AG (Accept Grammatical), AU (Accept Ungrammatical) and RU (Reject Ungrammatical) trials.

Our statistical analysis, which follows Huang and Staub (2021), directly addresses two questions. First, is there disruption on the trials on which readers accept an ungrammatical sentence, compared to when they accept a grammatical sentence? Second, is there disruption on the trials on which readers reject an ungrammatical sentence, compared to when they accept an ungrammatical sentence? To carry out our analyses, we first excluded the small number of trials on which subjects rejected a grammatical sentence. We then labeled the remaining trials as accept grammatical (AG), accept ungrammatical (AU), and reject ungrammatical (RU). Figure 3 shows the by-subject means and standard errors for each eye movement measure, for each region, as well as total sentence reading time, for each of these three subsets of trials. (Note that the RU means do not reflect data from all subjects; as shown in Figures 1 and 2, several subjects rejected none of the ungrammatical sentences.)

In our statistical models, we coded these three levels with backward difference contrasts. The reference level was AG, the first contrast compared AU to AG, and the second compared RU to AU. For each reading time measure on each region, as well as for total sentence reading time, we computed Bayesian linear mixed-effects models with log transformed reading time as the dependent variable, using the *brms* package (Bürkner, 2017) in the R statistical programming environment (version 4.4.3). For regressions out, we computed a Bayesian mixed-effects logistic regression model. Each model included random by-subject and by-item intercepts, as well as random slopes by subjects and items for each of the two contrasts. For all models, we use the default priors implemented in *brms*; for the fixed effects, these are flat priors, which result in posterior distributions that are determined primarily by the likelihood rather than the prior. Each chain ran for 4000 iterations. For all parameters of all models, Rhat was no greater than 1.01.

Table 1 reports the model estimates for the AG level (i.e., the model’s intercept), as well as the estimate for each contrast (the mean of the posterior distribution), the lower and upper bounds of the 95% credible interval, and the proportion of the posterior distribution that is in the same direction as the mean, computed using the *p_direction* function from the *bayestestR* package (Makowski et al., 2019). As a heuristic, we treat an effect as credible when the probability on one side of 0 is greater than .975.

**Table 1.** Experiment 1 model estimates. AG: Accept Grammatical; AU: Accept Ungrammatical; RU: Reject Ungrammatical. See text for model details. Effects with *p*(direction) > .975 are in **bold**.

	**Estimate**	**Lower 95% CI**	**Upper 95% CI**	***p*(direction)**
**Pre-Target Region**				
First Pass Time				
AG	5.45	5.40	5.49	
AU-AG	0.02	−0.02	0.06	.89
RU-AU	0.03	−0.05	0.11	.79
Go-Past Time				
AG	5.74	5.67	5.82	
AU-AG	0.01	−0.04	0.06	.65
RU-AU	0.05	−0.05	0.15	.83
Total Time				
AG	5.93	5.84	6.02	
AU-AG	0.01	−0.04	0.06	0.66
RU-AU	**0.29**	**0.18**	**0.40**	**1**
Regressions				
AG	−1.21	−1.50	−0.94	
AU-AG	−0.03	−0.27	0.20	.62
RU-AU	0.04	−0.47	0.51	.58
**Target Region**				
First Pass Time				
AG	5.83	5.76	5.90	
AU-AG	**0.05**	**0.01**	**0.09**	**.99**
RU-AU	0.05	−0.02	0.12	.92
Go-Past Time				
AG	6.08	5.99	6.16	
AU-AG	0.03	−0.02	0.09	.90
RU-AU	**0.13**	**0.01**	**0.24**	**.98**
Total Time				
AG	6.39	6.29	6.48	
AU-AG	**0.04**	**0.00**	**0.09**	**.98**
RU-AU	**0.26**	**0.17**	**0.35**	**1**
Regressions				
AG	−1.70	−1.97	−1.46	
AU-AG	0.07	−0.20	0.34	.67
RU-AU	0.44	−0.05	0.88	.96
**Final Region**				
First Pass Time				
AG	6.12	5.97	6.26	
AU-AG	0.01	−0.05	0.06	.60
RU-AU	**−0.15**	**−0.25**	**−0.04**	**1**
Go-Past Time				
AG	7.31	7.17	7.45	
AU-AG	0.03	−0.03	0.08	.83
RU-AU	**0.26**	**0.12**	**0.41**	**1**
Total Time				
AG	6.57	6.40	6.73	
AU-AG	0.01	−0.03	0.05	.64
RU-AU	0.00	−0.10	0.11	.53
Regressions				
AG	0.77	0.46	1.08	
AU-AG	0.04	−0.17	0.25	.67
RU-AU	**0.62**	**0.16**	**1.12**	**1**
**Total Sentence Reading Time**				
AG	8.30	8.21	8.40	
AU-AG	−0.01	−0.03	0.02	.66
RU-AU	**0.17**	**0.11**	**0.24**	**1**

On the pre-target region, there was little evidence that reading times or regressions differed for AU compared to AG. For the comparison of RU and AU, the only credible effect was increased total time for RU; back transformation of the model estimate indicates an effect of about 127 ms.

On the target region, AU demonstrated a credible difference from AG in both first pass time, by about 18 ms, and total time, by about 24 ms. There was no credible go-past effect, nor was there an effect on the regressions measure. RU demonstrated longer go-past time than AU, by about 61 ms, as well as longer total time, by about 153 ms, and an increase in the probability of regression, by about 7% (though the latter effect had a probability of direction of only .96).

On the final region, AU did not credibly differ from AG on any measure. However, RU did credibly differ from AU in first pass time, go-past time, and regression probability. First pass time was actually 64 ms *shorter* for RU than for AU, while go-past time was about 440 ms longer, and the probability of a regression increased by about 12%. Thus, it appears that first pass time was truncated in the RU trials when readers initiated a regression.

Finally, total sentence reading time did not credibly differ between AU and AG trials. On the other hand, total sentence reading time for RU trials was credibly longer than in AU trials, by about 746 ms.

### Discussion

Subjects detected the agreement errors in this experiment relatively rarely; the mean detection rate was 21.7%, and the median subject rejected only three out of twenty ungrammatical sentences. Though it is well known that the presence of a plural local noun reduces the rate at which readers judge sentences with a singular subject and a plural verb to be unacceptable, the rejection rate in the present experiment is, we believe, the lowest that has been reported in the literature. The rejection rate for the intermixed implausible double-object sentences indicates that subjects were indeed engaged with the task, and the results of the post-experiment questionnaire, given to 28 subjects, indicate that our subjects do generally judge these agreement violations to be ungrammatical when asked to make an explicit judgment of a sentence that is fully visible during the judgment process. We infer that the explicit judgment tasks that are typically used in the literature overestimate the salience of these agreement violations in the course of normal reading.

Turning to the eye movement data, on the trials on which subjects did correctly reject the sentences containing an agreement violation, reading times were much longer than when they incorrectly accepted these sentences. Readers showed increased rates of regressive eye movements from both the target region and the final region of the sentence, much longer go-past reading times on both regions, and much longer total sentence reading times. This is not surprising; it is expected that readers’ eye movements would show evidence of disruption when they recognize that the sentence is ungrammatical, compared to when they do not.

The theoretically interesting comparison is between trials when subjects accepted the ungrammatical sentences and when they accepted the corresponding grammatical sentences. We see only very modest signs of disruption in reading the ungrammatical sentences, appearing in first pass time and total time on the target region, on the order of 20 ms. No regression-based measure showed disruption, and there was no disruption in total sentence reading time. It is especially worth noting that while the model estimated total sentence reading time to be 746 ms longer in RU trials than AU trials, it estimated total sentence reading time to be essentially identical in AU trials and in AG trials.

On their surface, these results seem to imply that even when the error is not noticed, the difficulty of agreement computation in the ungrammatical sentences does have an effect, though a very small and transitory one. But as noted in the Introduction, some difference in reading time between the two conditions might be expected based simply on the difference in the predictability of the plural verb. Are the small effects on the target region consistent with this difference in predictability? Before addressing this question, we present Experiment 2, which was a near-replication of Experiment 1.

## EXPERIMENT 2

Experiment 2 was identical to Experiment 1 except that the 40 judgment trials from the separate experiment with prepositional dative and double object sentences were removed, and these were replaced with 20 judgment trials that contained simple agreement errors. In half of these, a singular verb followed a plural subject, and in half a plural verb followed a singular subject. Examples are in (10) and (11):

(10) *Before the war, the ships was used for commercial transport.
(11) *The driveway were slick from the freezing rain that fell during the night.

We predicted that adding these simple agreement violations, which we assumed would be highly salient and would usually elicit a rejection of the sentence, would reduce readers’ bias to treat sentences as grammatical (Hammerly et al., 2019). We expected that this would result in substantially more rejections of the ungrammatical critical trials. In addition to testing this prediction, Experiment 2 provided an opportunity to replicate the eye movement results from Experiment 1.

### Methods

#### Participants.

Seventy-four subjects from the same subject pool as Experiment 1 completed the experiment, of whom five were excluded due to technical problems with their data files. None were excluded based on poor performance on the comprehension questions. Thus, final *n* = 69 (43 female, 26 male, age range from 18 to 34 years, median = 19).

#### Materials.

All items were identical to Experiment 1, except that, as noted above, the 40 judgment trials from a separate sub-experiment were replaced with 20 judgment trials containing simple agreement errors. Thus, each subject read 160 trials in total rather than 180, with 60 of these being judgment trials, and of the 60 judgment trials, 40 contained agreement violations.

#### Procedure.

The procedure was identical to Experiment 1.

### Results

All subjects all achieved at least 79% correct on the filler comprehension questions, with a mean of 92.7%. In total, 3 out of 2760 critical trials were lost due to technical errors, leaving 2757 critical trials available for analysis. As expected, the 20 simple agreement errors were highly salient; mean rejection rate for these items was 82.0%, with median of 90.0%.

**Figure 4.**
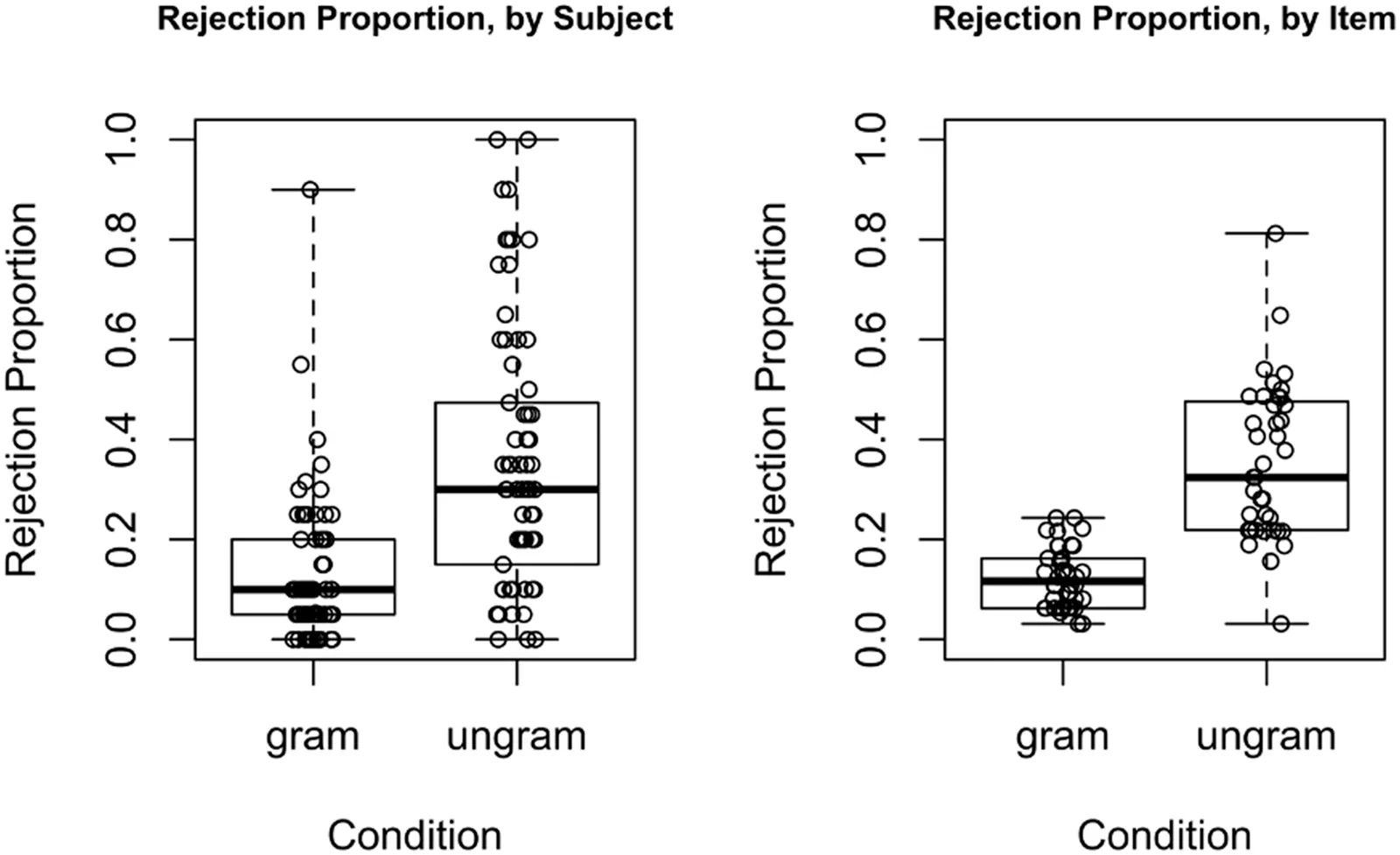
Proportion of sentences rejected in each condition in Experiment 2, by subject (left) and by item (right).

**Figure 5.**
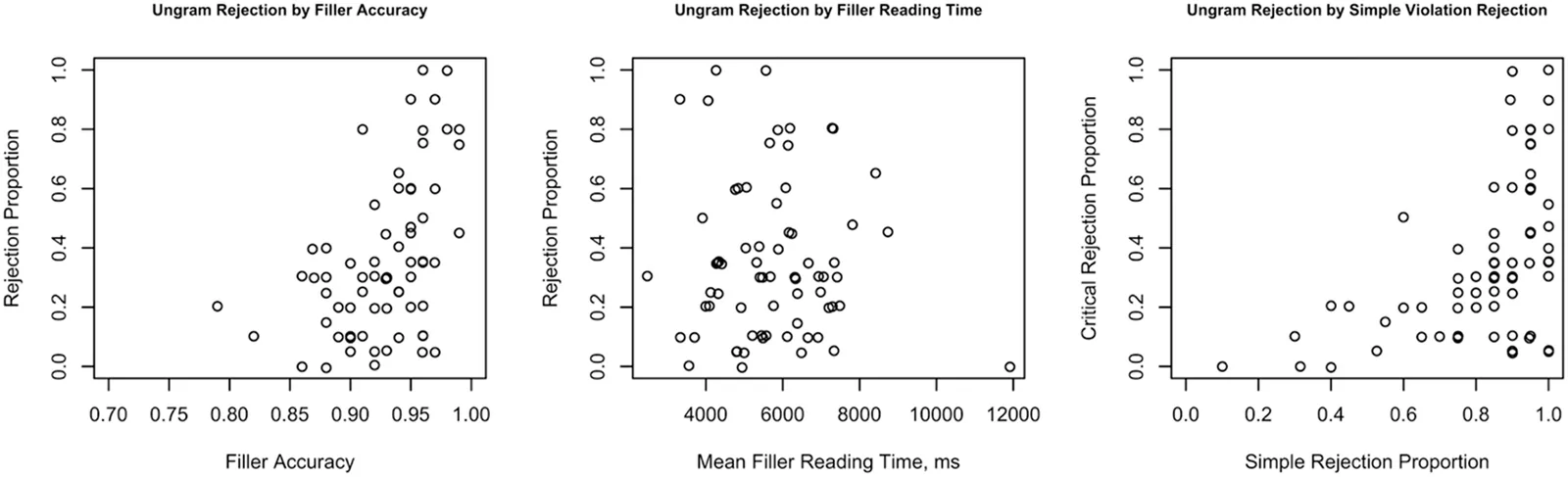
Experiment 2 relationship between filler comprehension accuracy and error detection rate in the critical sentences (left), between filler reading time and error detection rate in the critical sentences (center), and between rejection rate of the simple agreement violations and error detection rate in the critical sentences (right).

The inclusion of the simple agreement errors did appear to have the expected effect of increasing rejection of the agreement errors in the critical trials; below we directly assess differences between experiments. The mean rejection rate for these trials was 35.5% (compared to 21.7% in Experiment 1), and the median was 30% (compared to 15% in Experiment 1). The mean rejection rate for the grammatical condition also increased somewhat, from 8.5% to 12.5%. Figure 4 shows the proportion of trials that were rejected in each condition, by subject and by item. Figure 5 shows the relationship between subjects’ filler sentence reading time and rejection of the critical agreement violations, between filler sentence accuracy and rejection of these violations, and between rejection of the simple agreement errors and rejection of the critical violations. Like in Experiment 1, filler accuracy was positively correlated with rejection rate (*r* = .48, *p* < .001), but unlike in Experiment 1, this correlation did not hold for filler reading time (*r* = .03, *p* = .68). Subjects’ rejection of the simple agreement errors and their rejection of the critical agreement violations were also correlated (*r* = .50, *p* < .001).

**Figure 6.**
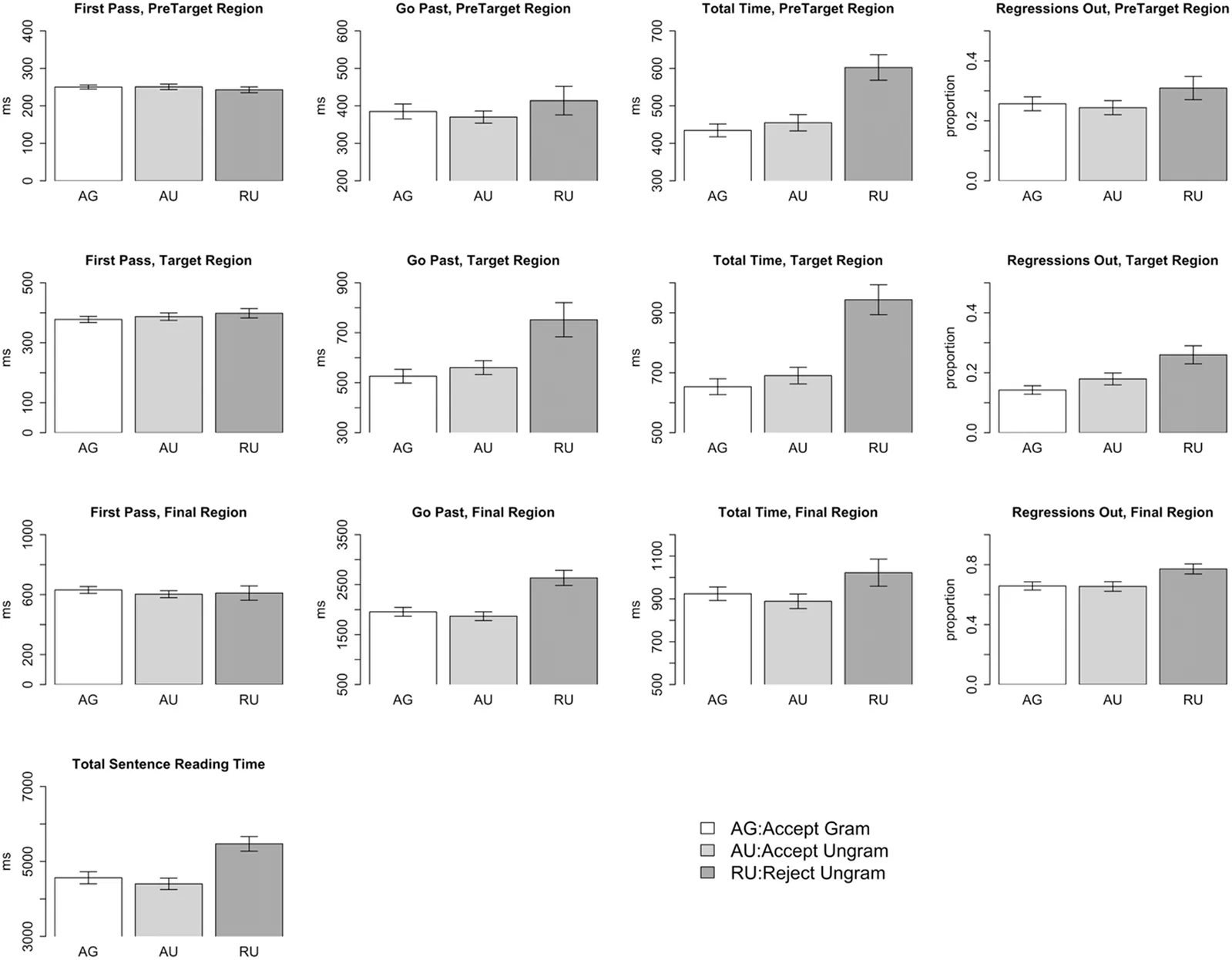
Experiment 2 mean and standard error, by subject, for each eye movement measure on each region, and for total sentence reading time, for AG (Accept Grammatical), AU (Accept Ungrammatical) and RU (Reject Ungrammatical) trials.

We turn now to analysis of reading times; all analysis procedures were identical to Experiment 1. Again, a trial was excluded from analysis of a region if that region was skipped. The pre-target region was skipped on 16.7% of trials in the grammatical condition, and 18.0% in the ungrammatical condition; the target region was skipped on 4.0% of trials in the grammatical condition and 3.3% in the ungrammatical condition; and the final region was skipped on 3.7% of trials in the grammatical condition and 4.1% in the ungrammatical condition. Figure 6 shows the by-subject means and standard errors for each reading time measure on each region, as well as total sentence reading time, for AG, AU, and RU trials. Table 2 provides the model estimates.

**Table 2.** Experiment 2 model estimates. AG: Accept Grammatical; AU: Accept Ungrammatical; RU: Reject Ungrammatical. See text for model details. Effects with *p*(direction) > .975 are in **bold**.

	**Estimate**	**Lower 95% CI**	**Upper 95% CI**	***p*(direction)**
**Pre-Target Region**				
First Pass Time				
AG	5.42	5.38	5.47	
AU-AG	−0.01	−0.05	0.04	.61
RU-AU	−0.01	−0.07	0.05	.63
Go-Past Time				
AG	5.71	5.64	5.78	
AU-AG	−0.05	−0.11	0.01	.93
RU-AU	0.06	−0.04	0.15	.89
Total Time				
AG	5.97	5.89	6.05	
AU-AG	0.01	−0.05	0.08	.66
RU-AU	**0.25**	**0.15**	**0.36**	**1**
Regressions				
AG	−1.26	−1.53	−1.00	
AU-AG	−0.19	−0.48	0.10	.90
RU-AU	0.20	−0.23	0.60	.83
**Target Region**				
First Pass Time				
AG	5.83	5.77	5.89	
AU-AG	−0.00	−0.04	0.04	.50
RU-AU	0.05	−0.01	0.11	.94
Go-Past Time				
AG	6.11	6.02	6.21	
AU-AG	**0.06**	**0.00**	**0.12**	**.98**
RU-AU	**0.17**	**0.05**	**0.29**	**1**
Total Time				
AG	6.43	6.35	6.52	
AU-AG	**0.07**	**0.01**	**0.12**	**.99**
RU-AU	**0.30**	**0.21**	**0.39**	**1**
Regressions				
AG	−1.63	−1.88	−1.38	
AU-AG	0.29	−0.03	0.62	.96
RU-AU	**0.63**	**0.25**	**0.98**	**1**
**Final Region**				
First Pass Time				
AG	6.12	5.98	6.26	
AU-AG	−0.03	−0.09	0.03	.85
RU-AU	**−0.13**	**−0.23**	**−0.02**	**.99**
Go-Past Time				
AG	7.34	7.22	7.47	
AU-AG	−0.01	−0.08	0.06	.56
RU-AU	**0.22**	**0.08**	**0.38**	**1**
Total Time				
AG	6.57	6.41	6.73	
AU-AG	0.00	−0.05	0.05	.54
RU-AU	−0.06	−0.16	0.14	.87
Regressions				
AG	1.04	0.74	1.36	
AU-AG	0.02	−0.24	0.27	.57
RU-AU	**0.55**	**0.14**	**1.02**	**1**
**Total Sentence Reading Time**				
AG	8.36	8.27	8.45	
AU-AG	−0.02	−0.05	0.02	.82
RU-AU	**0.17**	**0.11**	**0.23**	**1**

As in Experiment 1, on the pre-target region neither reading times nor regressions credibly differed for AU compared to AG. Also as in Experiment 1, for the comparison of RU and AU on the pre-target region, the only credible effect was increased total time for RU; the estimate of the difference between RU and AU is approximately 111 ms.

On the target region, unlike in Experiment 1, AU trials did not show longer first pass time than AG trials; indeed, the sign of the parameter estimate was very slightly negative. However, go-past time was longer for AU trials; the model estimate corresponds to an effect of about 28 ms. As in Experiment 1, there was also an effect in total time, which was about 45 ms longer in AU trials. There was also a hint of an effect on regressions, though it did not reach the criterion of *p*(direction) > .975. For the comparison of RU and AU trials on the target region, it was again the case the RU trials showed longer reading times and more regressions across the board, with the exception that the first pass effect did not reach the *p*(direction) threshold of .975. The effects on go-past time and total time correspond to effects of 83 ms and 217 ms, respectively. The effect on regressions corresponds to a difference of about 14%.

On the final region, as in Experiment 1 there were no credible differences between AU and AG trials. Also like in Experiment 1, first pass time was shorter for RU trials than AU trials, and RU and AU trials did not credibly differ in total time. However, once again go-past time was much longer in RU trials than AU (by 379 ms), and there were about 9% more regressions.

Finally, total sentence reading time did not differ between AU and AG trials, while total sentence reading time was much longer in RU trials than AU trials; the estimate of this effect is about 792 ms.

### Discussion

Including simple agreement errors in Experiment 2 increased the rate at which readers rejected the critical agreement violations, to 35.5%. As in Experiment 1, readers demonstrated much longer reading times when they rejected the critical ungrammatical sentences than when they did not. In both experiments, the estimate of the effect on total sentence reading time was over 700 ms, and was due to extensive rereading following regressions from both the target region and the final region, as shown by the increased go-past time on both regions, as well as increased total time on the pre-target region.

Also as in Experiment 1, readers did not demonstrate global difficulty in AU trials compared to AG trials. In both experiments, total sentence reading time was not longer in AU trials than in AG trials; indeed, the sign of the parameter estimate was negative in both experiments. No effects reached significance on either the pre-target region or the final region. However, as in Experiment 1, there was evidence of a small but credible increase in reading time on the target region in AU trials. In Experiment 1 this effect was seen in first pass time and total time, while in Experiment 2 it was seen in go-past time and total time.

In summary, the eye movement results from the two experiments are extremely similar, with the one notable difference being the specific target region eye movement measures in which an AU-AG difference appeared. As we have noted, it is necessary to address the possibility that the AU-AG difference on the target region can be attributed to lexical predictability. Before turning to that analysis, we present a combined statistical analysis of the two experiments. The goals of this analysis are, first, to ascertain which eye movement effects are credible with the increased power of the combined data, and second, to determine whether any effects differ between the two experiments.

## ANALYSIS OF COMBINED EXPERIMENTS

We begin by analyzing the judgment responses for the combined data. We constructed a mixed-effects Bayesian logistic regression model with grammaticality, experiment, and their interaction as fixed effects; both grammaticality and experiment were sum coded, with the levels −.5, .5. Random subject and item intercepts were included, as well as random by-subject and by-item slopes for the effect of grammaticality. This model obtained a credible effect of grammaticality on the probability of an ‘accept’ response (*p*(direction) = 1) as well as a credible effect of experiment (*p*(direction) = 1). However, the hint of an interaction—i.e., a larger effect of grammaticality in Experiment 2 than in Experiment 1—was not fully credible (*p*(direction) = .91). Thus, including the simple agreement errors in Experiment 2 resulted in an increase in ‘reject’ responses for both grammatical and ungrammatical critical sentences; we do not have statistical evidence that this increase was larger for ungrammatical sentences.

For analysis of the combined eye movement data, we focus only on the target region, as the results for the pre-target and final regions are extremely similar for Experiments 1 and 2. We constructed Bayesian statistical models with the same contrast coding scheme used above (AG as intercept, and AU-AG and RU-AU as the two contrasts), but with the additional fixed effect of Experiment, sum coded with −.5, .5, and with the interactions of Experiment and each of the two contrasts; in these models, the intercept reflects AG trials averaged across the two experiments, while the main effect of Experiment reflects the difference between the two experiments for AG trials. We again included subject and item intercepts, as well as by-subject and by-item slopes for the critical contrasts. The results of these models are shown in Table 3.

**Table 3.** Model estimates for target region eye movement measures for combined data from Experiments 1 and 2. AG: Accept Grammatical; AU: Accept Ungrammatical; RU: Reject Ungrammatical. See text for model details. Effects with *p*(direction) > .975 are in **bold**.

	**Estimate**	**Lower 95% CI**	**Upper 95% CI**	***p*(direction)**
First Pass Time				
Intercept	5.83	5.78	5.88	
Experiment	−0.00	−0.08	0.07	.53
AU-AG	0.02	−0.01	0.05	.95
RU-AU	**0.05**	**0.00**	**0.10**	**.99**
Experiment × AU-AG	−0.05	−0.10	0.01	.95
Experiment × RU-AG	0.00	−0.08	0.09	.54
Go-Past Time				
Intercept	6.09	6.02	6.17	
Experiment	0.04	−0.06	0.14	.81
AU-AG	**0.05**	**0.01**	**0.08**	**.99**
RU-AU	**0.15**	**0.07**	**0.22**	**1**
Experiment × AU-AG	0.03	−0.05	0.10	.75
Experiment × RU-AG	0.07	−0.06	0.21	.86
Total Time				
Intercept	6.41	6.34	6.49	
Experiment	0.05	−0.07	0.16	.79
AU-AG	**0.05**	**0.02**	**0.09**	**1**
RU-AU	**0.28**	**0.22**	**0.35**	**1**
Experiment × AU-AG	0.03	−0.04	0.09	.78
Experiment × RU-AG	0.03	−0.09	0.15	.68
Regressions				
Intercept	−1.65	−1.85	−1.45	
Experiment	0.07	−0.22	0.36	.68
AU-AG	0.18	−0.03	0.39	.96
RU-AU	**0.54**	**0.26**	**0.81**	**1**
Experiment × AU-AG	0.28	−0.09	0.65	.93
Experiment × RU-AG	0.18	−0.31	0.68	.77

There were no credible effects of Experiment, nor were there credible interactions between Experiment and either of the two contrasts. Thus, we have no statistical evidence that results from the two experiments differ in any respect on the target region. In the combined data, the RU-AU contrast is credible for all four measures, as would be expected based on the results from each of the two individual experiments. The AU-AG difference is not credible for first pass time (with the estimate corresponding to a 7 ms effect) or for regressions. However, this difference is credible for go-past time and total time; the go-past effect in the combined data is about 23 ms, while the total time effect is about 31 ms.

## SURPRISAL GENERALIZATION ANALYSIS

Our combined analysis revealed evidence for an ungrammaticality-related slowdown in accepted trials, i.e., a modest difference between AU and AG trials in both go-past and total time. A critical question is whether this slowdown is of a magnitude that could be accounted for by the difference in the surprisal of the verb between the two conditions, or if it is larger than expected based on this difference. In other words: Is there evidence that the slowdown observed in AU compared to AG trials reflects a cost beyond what is expected due to surprisal? To address this question, we performed a surprisal generalization analysis inspired by van Schijndel and Linzen (2021) and Huang et al. (2024). This analysis leverages the data from the filler items (not including the implausible/ungrammatical items that also had judgment questions) from both experiments, to build a statistical model of the effect of surprisal on different reading time measures in our experiment, and then uses this model to generate quantitative predictions for a surprisal-related slowdown in the AU-AG contrast in the combined data.

To quantify surprisal, we used an English-language LSTM language model from Gulordava et al. (2018)^3^. We extracted in-context surprisal values for all the words in our 100 filler sentences, along with the surprisal of the words in the critical region in our target stimuli. When a single orthographic word corresponded to more than one subword token in the language model, the surprisals of the constituent subword tokens were summed. The average surprisal of the critical auxiliary was 2.5 bits in the grammatical condition, corresponding to a probability of approximately .18 (ranging between 1.3 and 4.8 bits across items), and 5.6 bits in the ungrammatical condition, corresponding to a probability of approximately .02 (ranging between 3.1 and 9.0 bits). The average surprisal of the following participial was 5.5 bits in the grammatical condition (ranging between 1.5 and 10.9 bits) and 5.7 bits in the ungrammatical condition (ranging between 2.0 and 11.3 bits). Thus, we expected that any differences between conditions associated with surprisal should be driven by differences in the surprisal of the auxiliary itself.

Next, we implemented a Bayesian variant of van Schijndel and Linzen (2021)’s methodology.^4^ A full specification of this model can be found in Appendix A. The goal of this analysis is to provide an estimate of the *predicted agreement cost*, or the difference between reading times in the Accepted Ungrammatical and Accepted Grammatical conditions in our experiment (the AU-AG contrast).

To estimate this cost, we follow van Schijndel and Linzen in estimating a surprisal-to-RT conversion model from our fillers. These models were implemented as Bayesian hiearchical linear regressions, fitted to the combined eye movement data on first-pass, go-past, and total reading times from both experiments (for full details of the fitted models, see Appendix A). To prevent the influence of wrap-up effects, we excluded the two final words of every filler sentence from this model (inspired by Smith & Levy, 2013). We modeled word-level reading times as a function of the surprisal of that word and the two preceding words, in addition to lexical-level control predictors (centered and scaled log frequency and length of the target word and two preceding words, as well as their interaction). Because raw RT is expected to scale linearly with surprisal, we modeled raw reading times for each measure (see Huang et al., 2024 for further discussion of the impact of this design choice). The fitted conversion factors for each model are presented in Table 4; the lexical control variables are omitted for space.

**Table 4.** Summaries of the posterior distribution over the intercept and surprisal coefficient estimates from the three Bayesian hierarchical regression models fitted to the filler data. Each model is fitted as described in Appendix A. The surprisal predictors for the target word (*w*_*i*_), preceding word (*w*_*i*−1_), and word before that (*w*_*i*−2_) are presented; all other predictors omitted for clarity. Coefficient values express the predicted slowdown, expressed in ms/bit, for each additional bit of surprisal on a given word.

	**Mean**	**95% Credible interval**
First Pass Time		
Intercept	238	[231, 245]
Surprisal *w*_*i*_	2.3	[1.8, 2.8]
Surprisal *w*_*i*−1_	1.0	[0.6, 1.3]
Surprisal *w*_*i*−2_	−0.6	[−0.9, −0.4]
Go-Past Time		
Intercept	315	[298, 333]
Surprisal *w*_*i*_	5.5	[4.1, 6.9]
Surprisal *w*_*i*−1_	7.0	[5.8, 8.1]
Surprisal *w*_*i*−2_	2.6	[1.7, 3.4]
Total Time		
Intercept	312	[298, 326]
Surprisal *w*_*i*_	8.0	[6.7, 9.4]
Surprisal *w*_*i*−1_	4.6	[4.0, 5.3]
Surprisal *w*_*i*−2_	2.0	[1.5, 2.5]

With the fitted surprisal-to-RT conversion models in hand, we defined the *agreement cost* as the predicted RT difference between the ungrammatical and grammatical conditions of a given experimental item, conditioned on the posterior distribution over the parameter estimates in our Bayesian surprisal-to-RT conversion models and the surprisal values for the grammatical and ungrammatical variants of a given item (see Appendix A for formal definition). This item-wise agreement cost was then averaged across all participants and items in our experiments, yielding the average predicted agreement cost for our experimental AU-AG contrast. In this way, we calculated the posterior distribution over the predicted agreement cost for first-pass, go-past, and total reading times. Although it was the surprisal of the auxiliary, not the participle, that differed across conditions, an agreement cost is predicted at both words because higher surprisal on a given word is associated with a slowdown in the spillover region in our data (Table 4).

Note that our models provide an estimate of the agreement cost at the level of the word, whereas our experimental analyses aggregate reading times on a two-word region consisting of the auxiliary and participial. The predicted agreement cost for each word is a prediction for trials in which that word was fixated rather than skipped; when a word was skipped in the filler trials, data from that word did not enter into the model’s estimate of the effect of surprisal. However, the observed reading times for the two-word region combine three distinct types of trials: (a) cases in which the auxiliary was fixated, but the participial was not; (b) cases in which the participial was fixated, but the auxiliary was not; and (c) cases in which both words were fixated. Thus, the observed agreement effect on reading times for the two-word region is not directly comparable to the sum of the predicted agreement costs for the two individual words; the expected relationship between the observed cost and the predicted costs for the individual words will depend on, e.g., whether on trials on which the reader fixates both words, a cost is paid on both fixations, or on only one. Therefore, we provide three estimates of the predicted agreement cost for each eye-tracking measure: The predicted agreement cost for the auxiliary, the predicted agreement cost for the participial, and the predicted agreement cost based on the summed surprisal of the full region (see Appendix A). The individual agreement costs for the auxiliary and participial words may be interpreted as a lower bound estimate of the AU-AG contrast, and the agreement cost for the full region may be interpreted as an upper bound estimate of the AU-AG contrast. This is because they correspond, respectively, to the expected RT slowdowns if readers pay an agreement cost on only one word or on both words in our critical region.

These predicted effect sizes were then compared against the estimated empirical effect size in the combined dataset. The effect size was estimated using a Bayesian mixed-effects regression on reading times for each measure, with a simplified model fitted just to the accepted trials with a single predictor for grammaticality. However, for the purposes of this analysis we fitted raw as opposed to log transformed reading times as the dependent measure; raw reading times were used here because the predicted effect sizes were also generated from a model based on raw reading times.

**Figure 7.**
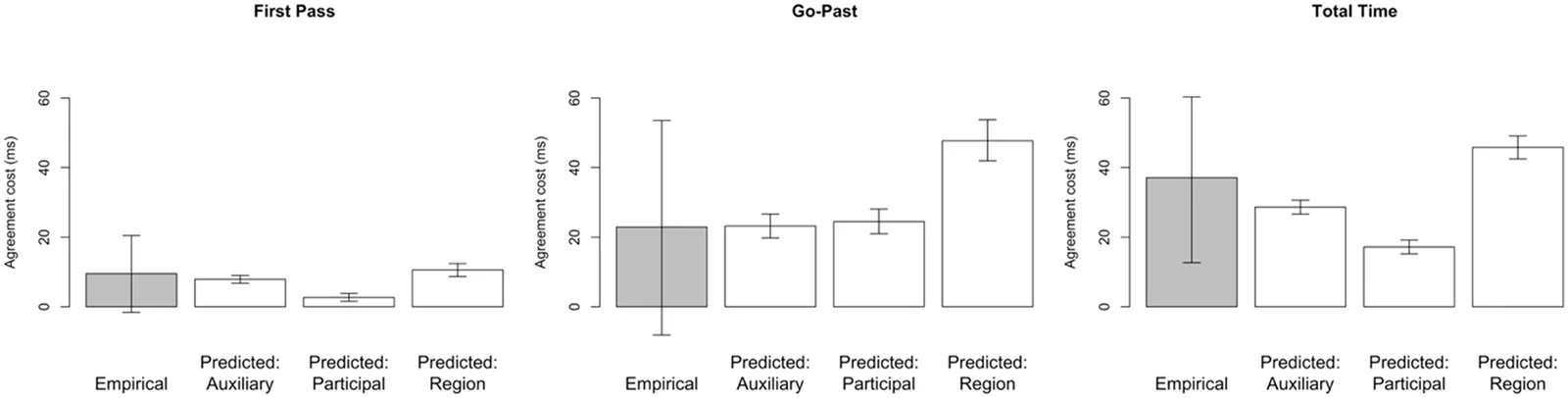
Observed and predicted agreement costs effects for accepted trials in the combined E1–E2 dataset, in each of first-pass, go-past, and total reading times. Observed costs (in grey) plot the mean and 95% credible interval on the estimated agreement cost (AU-AG contrast). Predicted agreement costs (in white) plot the mean and 95% credible interval of the agreement cost at the auxiliary, participial, and aggregated region (see Appendix A).

Figure 7 displays the main results^5^. For all three measures, we observe considerably more uncertainty in the empirical effect size than there is in the predicted effect size. Nevertheless, it is clear that there is a fairly close alignment between the observed agreement costs (at the level of the region) and the predicted agreement costs for the auxiliary itself. On the other hand, the mean predicted agreement cost for the full region is generally is larger than the mean observed agreement cost.

Although the close alignment between the means of the predicted and observed agreement costs is suggestive, the significant uncertainty in the observed estimates precludes any strong conclusions about equivalence between predicted and observed costs. However, it does suggest an answer to the main question we sought to answer with this analysis: *Is there evidence that the slowdown observed in AU compared to AG trials reflects a cost beyond what is expected due to predictability?* The results of this analysis suggest that answer is ‘*no*.’ The observed empirical cost is never clearly greater than the predicted cost; the predicted reading-time slowdown due to the surprisal does not underpredict the observed processing difficulty. In this respect, the present analysis diverges distinctly from similar analyses on different datasets (Arehalli et al., 2022; Huang et al., 2024; van Schijndel & Linzen, 2021; Wilcox et al., 2021).

## GENERAL DISCUSSION

In two experiments, we used the IJP to explore the relationship between incremental processing difficulty as revealed in the eye movement record and the conscious detection of agreement errors. We summarize the empirical findings before turning to their theoretical implications.

The judgment data revealed that in the IJP setting, comprehenders recognize ungrammatical agreement in agreement attraction configurations at a very low rate: 21.7% in Experiment 1 and 35.5% in Experiment 2. In Experiment 2, where filler trials with simple agreement violations were included, the rejection rate for grammatical control sentences also increased, from 8.5% to 12.5%.

Analysis of the eye movement record revealed substantial disruption in the reading of ungrammatical sentences when comprehenders did later report an error. In the combined analysis of the critical region of Experiments 1 and 2, rejected ungrammatical trials were associated with longer reading times than accepted ungrammatical trials in first pass time, go-past time, and total time, and also induced more regressions. Rejected ungrammatical trials also induced much longer total sentence reading times, in both experiments. These were sizable effects: Total reading time on the critical region was inflated by over 150 ms when an ungrammatical sentence was rejected, and total sentence reading time was inflated by over 700 ms.

When comparing sentences with ungrammatical agreement that were ultimately accepted to sentences with grammatical agreement that were accepted, we observed much smaller reading time effects on the critical region, which were credible in go-past and total time in the combined analysis. A surprisal generalization analysis (e.g., van Schijndel & Linzen, 2021) revealed that the slowdown in the ungrammatical accepted trials is no larger than what would be expected given the differences in the surprisal of the critical verb across the two conditions. There were no credible effects on other regions, or in the total reading time measure.

### Agreement Processing, Bias, and the Role of the Task

We begin by discussing the implications of the acceptability judgment results. The rate at which agreement attraction errors are noticed when acceptability judgment is not the primary explicit task is far lower than might be expected. Comprehenders noticed an agreement error in Experiment 1 in only 21.7% of ungrammatical sentences. While Wagers et al. (2009) reported a 45% rejection rate for similar sentence types in a speeded binary grammaticality judgment task, we do not know of any study with a rejection rate as low as the rate obtained in Experiment 1. But the rate at which errors are detected in the IJP is also impacted by the salience and/or prevalence of other agreement errors in the experiment: The rejection rate increased to 35.5% in Experiment 2, when an additional twenty sentences with simple agreement errors were included.

These results illustrate the critical role of decisional parameters in acceptability judgment tasks. Both of the critical observations—the low rate of rejection of these sentences in Experiment 1, and the increase in Experiment 2—are consistent with the proposal by Hammerly et al. (2019) that judgments of the acceptability of subject-verb agreement involve two critical parameters: (a) a continuously-valued representation of subject number, which is affected by the local noun’s number; and (b) the participant’s bias to respond ‘grammatical’ in the decision task. In the terms of Hammerly et al.’s evidence accumulation model, the bias parameter determines the relative quantity of evidence that is required in order for the participant to conclude that there is a number match or mismatch between subject and verb. Hammerly et al. observed a substantial bias toward a ‘grammatical’ response in a standard judgment task, but also observed that this bias could be reduced or even eliminated by making the majority of sentences in an experimental session ungrammatical.

Interpreting the present data through the lens of the Hammerly et al. model, the very low rate at which agreement errors were detected in Experiment 1 reflects a strong bias towards a ‘grammatical’ response. Such a bias would be expected, for two reasons. First, a strong bias toward grammaticality may generally be present in the IJP, where the main task is presented as reading for comprehension, as opposed to checking the well-formedness of each sentence. Second, in Experiment 1 the twenty critical trials with agreement errors were the only genuinely ungrammatical sentences out of the 180 trials in the experiment. In Experiment 2, the addition of 20 very salient agreement violations would then have decreased subjects’ bias toward grammaticality. Notably, a bias-oriented account predicts that the rate of rejection might also increase in the grammatical condition in Experiment 2, which it did; statistically, there was a main effect of experiment on the rate of rejection, a main effect of condition, and no interaction.

Laurinavichyute and von der Malsburg (2024) presented a recent demonstration of the role of task demands in agreement attraction that dovetails with the present results. These authors focused on agreement attraction effects in grammatical sentences; the effect of the local noun’s number would be to potentially reduce the acceptability of a grammatical sentence, rather than to increase the acceptability of an ungrammatical sentence. In an experiment in which subjects expected to make a numerical acceptability rating after reading the critical grammatical sentences, there was an effect of the local noun’s number on these numerical ratings. But in a separate experiment in which subjects expected only comprehension questions, but were then asked to rate acceptability as well, this effect was absent altogether; notably, an effect on reading times was also eliminated when subjects expected comprehension rather than judgment questions. Laurinavichyute and von der Malsburg interpreted these results as reflecting the role of grammaticality bias, as proposed by Hammerly et al. (2019); they write, “The acceptability rating but not the question answering task prepares participants for encountering ill-formed sentences” (p. 14). We make a similar observation with regard to the IJP: The task is presented as a comprehension task, and participants are only occasionally asked to make a judgment, so they are generally not expecting ill-formed sentences. In sum, the present results, together with previous results presented by Hammerly et al. (2019) and Laurinavichyute and von der Malsburg (2024), illustrate that whether agreement is judged to be grammatical may depend heavily on the task context. We assume that judgments of other sentence types may also be modified by changing the task context and the nature of the filler trials.

### Theoretical Consequences of Our Eye Movement Results

We now turn to the implications of the eye movement results. As noted in the Introduction, we assume that ‘integration failure,’ in E-Z Reader 10’s sense, leads to a report of an error in the IJP. If this linking hypothesis is correct, then the present results straightforwardly support the E-Z Reader 10 post-lexical processing mechanism, whereby such processing impacts the forward progression of the eyes only when there is integration failure, or more generally, when an error is detected in the unfolding linguistic stimulus. On trials where no error is reported—and hence, by hypothesis, no integration failure has occurred—the localized, modest increase in reading time of the critical region is entirely predicted by differences between conditions in the predictability of the verb. Within the E-Z Reader framework, effects of predictability are understood as effects on lexical, not post-lexical, processing; other eye movement models (e.g., Engbert et al., 2005) make similar assumptions. This is consistent with interpretations of the effect of surprisal that emphasize its utility for efficient perceptual processes and optimally efficient word recognition (Smith & Levy, 2013). On the other hand, when an error is reported—when, by hypothesis, an integration failure has occurred—reading disruption is much more pronounced than is predicted by differences in predictability, and is characterized by an increase in regressive eye movements. In short, there seem to be two qualitatively distinct types of trials in our data: trials where integration fails and difficulty is pronounced, and trials where integration is successful and the small reading time effect is explicable on the basis of lexical predictability.

Our surprisal generalization results stand in stark contrast to the results of other surprisal generalization analyses (Arehalli et al., 2022; Huang et al., 2024; van Schijndel & Linzen, 2021; Wilcox et al., 2021), which have generally found that surprisal substantially *underpredicts* the processing costs associated with various structural processing phenomena, including the processing of mismatched verbal agreement (Huang et al., 2024; Slaats et al., 2026; see also Wilcox et al., 2021). A critical difference between these previous studies and the current study, however, is that the use of the IJP in the current study allowed sorting of trials into ‘reject’ and ‘accept’ trials. The surprisal generalization analysis we carried out suggests that surprisal only explains the magnitude of the observed processing difficulty in ‘accept’ trials. This conclusion is consonant with the more recent findings of Timkey et al. (2025), who showed that large language model surprisal has impressive predictive power for early eye movement measures on material disambiguating a garden-path, but only for those trials on which readers do not execute a regression at the critical region. This plausibly reflects an effect parallel to the one we see here: When no error is detected (or no regression is triggered), language model surprisal is able to predict almost all of the difficulty in reading, even in structurally complex sentences.

Our empirical conclusion that ungrammatical stimuli result in two categorically distinct kinds of trials in reading—failure on some occasions, success on others—is also consistent with a broader range of findings. For example, this conclusion is broadly consistent with the finding that regressive eye movements associated with ungrammaticality are associated with the P600 ERP component, an electrophysiological index of error detection or correction (Metzner et al., 2017). It is also consistent with the finding that the RT distribution of processing difficulty in syntactically challenging configurations is well modeled by a mixture distribution that posits multiple, categorically distinct types of trials (Paape & Vasishth, 2022). Indeed, Paape and Vasishth’s (2022) multinomial processing tree model explicitly proposes that the difficulty associated with garden pathing can be decomposed into the surprisal cost levied by disambiguating words (Hale, 2001; Levy, 2008) and an additional cost associated with the recognition and reanalysis of syntactic errors that impacts only a subset of trials. Interestingly, such a bimodal distribution is less evident in numerical acceptability judgment measures of garden path processing, raising the counterintuitive possibility that judgment measures, rather than online reading measures, more directly reflect the underlying processes (Dillon et al., 2019).

We conclude on the basis of the present results that there are two major underlying processes reflected in the eye movements associated with subject-verb agreement violations, and plausibly other structurally complex phenomena like garden paths. The first is predictability or lexical surprisal, the role of which in word-by-word processing is supported by a very large body of empirical results (e.g., Shain et al., 2024; Smith & Levy, 2013), and which plausibly reflects the centrality of prediction as a driving factor in language comprehension (Hale, 2001; Kuperberg & Jaeger, 2016; Levy, 2008; Ryskin & Nieuwland, 2023; Slaats & Martin, 2025; Staub, 2025). The second refers to the underlying cognitive processes that we might associate with EZ Reader’s notion of integration failure: a categorical but stochastic error signal that often leads to regressions and rereading, and is associated with conscious awareness of ill-formedness, which can be reported in a post-sentence judgment.

A range of underlying processes might contribute to such an error signal. It may simply be the outcome of a failure of normal processing mechanisms to reach a successful, coherent parse of the input. This could arise, for instance, if the beam width of a limited capacity parser simply lacks any analysis able to accommodate the current input, an outcome sometimes conceived of as ‘parser failure’ (Frazier & Rayner, 1982; Levy et al., 2008), or if the input is insufficiently harmonic or well-formed (Cho et al., 2017; Smith, 2018; Tabor et al., 1997; Wagers & Pendleton, 2015). But it could also reflect a more ‘active’ error monitoring mechanism, such as an online conflict detection mechanism (Kolk et al., 2003; Ness et al., 2025; van Herten et al., 2006), or an online ‘noisy channel’ error correction process (Bicknell & Levy, 2010; Gibson et al., 2013; Levy et al. 2009; Li & Ettinger, 2023; Li & Futrell, 2024; Ryskin et al., 2021). On this view, ‘integration failure’ in EZ Reader’s terms would represent a functionally significant error signal that triggers downstream conflict resolution or Bayesian inference mechanisms necessary to resolve the interpretation of the input. For example, on a ‘noisy channel’ account, the mismatched verbal agreement could lead to the probabilistic inference that the speaker had intended an alternative sentence where the subject of the sentence is plural, instead of singular (see Ryskin et al., 2021). A third and final possibility is that this reflects a simple ‘time out’ on the post-lexical processes that integrate a word into a larger sentence representation. As noted in the Introduction, Engelmann et al. (2013; following Bartek et al., 2011; Mitchell et al., 2008) proposed that integration failure reflects a time-out on an underlying processing operation, such as when the integration of word *n* has not been completed when the integration of word *n* + 1 has begun (see also Nicenboim & Vasishth, 2018). The present data do not let us cleanly arbitrate between these possibilities, but they do significantly limit the underlying syntactic processing mechanisms that we would expect to be reflected in eye movements. To wit, we expect eye movements to reflect discrete states of parser failure, parser time-out, or explicit error detection.

The present results dovetail in an interesting way with recent neurolinguistic results. For example, Stanojević et al. (2023) have shown that surprisal and structure-building networks can be dissociated in fMRI data, implying that they reflect distinct processes that can be spatially separated in the brain (see also Brennan & Pylkkänen, 2017). In a similar way, our own results show that behavioral measures can be decomposed into surprisal and a separate signal associated with processing ungrammaticality. What remains to be explored is whether there is any direct relationship between the various indices of structural processing explored in the neurolinguistic literature and the ‘failure’ or ‘error signal’ that seems to be present in the eye movement data.

Although the present results do not, on their own, implicate a unique underlying mechanism, a consideration of the wider literature makes both parser failure and explicit error detection tempting proposals. It is plausible that the mismatch between the agreement features of the verb and the subject in our stimuli could contribute to a drop in well-formedness or harmony of the overall structure that could drive an integration failure (e.g., Smith, 2018; Smith & Tabor, 2018). At the same time, there is evidence that mismatched agreement can trigger updates to the representation of the sentence, as expected on a ‘noisy-channel’ account (Ryskin et al., 2021): Keshev and Meltzer-Asscher (2021) and Molinaro et al. (2011) show that mismatched verbal agreement in Italian and Hebrew leads comprehenders to revise the number features of the subject, consistent with the view that error detection at the verb serves a functional role in allowing Bayesian inference processes to correct possible errors in the signal. And Keshev and Meltzer-Asscher (2021) show that agreement cues trade off probabilistically with other cues, like the probability of the underlying syntactic structures, in a way that is predicted by such an online ‘noisy-channel’ inference process.

One broader implication of our findings is that explicitly theorizing the conditions that contribute to failure, resulting in a signal to interrupt processing as usual, should be considered an integral part of any psycholinguistic theory. Importantly, this is in tension with intuitive linking theories that draw a simple alignment between fixation durations and the time cost of syntactic processing operations. It is common for researchers to predict a correlation between eye movement measures and the time cost of (say) retrieving a syntactic dependent (Lewis & Vasishth, 2005; Lewis et al., 2006) or resolving syntactic ambiguity (MacDonald et al., 1994; Trueswell et al., 1993), which implies an intuitive linking hypothesis in which the time cost of processing operations is directly reflected in eye movements (e.g., the eye-mind hypothesis of Just & Carpenter, 1980). However, as reviewed above, attempts to directly align processing theories with models of eye movements during reading typically do not feature such a transparent relationship between processing time and fixation durations (e.g., Engelmann et al., 2013; Nicenboim & Vasishth, 2018; Rabe et al., 2024; Reichle et al., 2009). The results presented here provide some general constraints on the alignment between processing theories and eye movement data during free reading.

### Implications for Theories of Agreement Attraction

In the Introduction, we described two current accounts of agreement attraction in comprehension: a continuous number valuation account (Hammerly et al., 2019), which posits that attraction arises because a subject phrase such as *The editor of the magazines* is represented as neither fully singular nor fully plural, and an account incorporating a cue-based retrieval mechanism for agreement checking (Engelmann et al., 2019; Wagers et al., 2009), which posits that attraction arises because the singular subject and the plural local noun each provides a partial match to a plural verb’s retrieval cues. Importantly, neither of these accounts makes explicit predictions about processing difficulty conditional on acceptance or rejection of a sentence. Hammerly et al. (2019) simulate predictions of an evidence accumulation model of a judgment task, where the continuously-represented value of the subject’s number determines the rate of evidence accumulation toward either a ‘grammatical’ or ‘ungrammatical’ judgment, and show that judgment latency is predicted to be generally slow in attraction configurations. However, this simulation does not explicitly distinguish RT for ‘grammatical’ and ‘ungrammatical’ judgments. Hammerly et al.’s Appendix A also provides a simulation of cue-based retrieval latency, showing that retrieval time with ungrammatical agreement is predicted to be very slow; see also the shiny app developed in association with Engelmann et al. (2019): https://engelmann.shinyapps.io/inter-act/. But again, these simulations do not distinguish between trials with ‘grammatical’ and ‘ungrammatical’ judgments. To our knowledge, there is no consensus regarding how to relate online memory retrieval processes and offline grammaticality judgments, making it difficult to specify the theory’s predictions about behavior in the two types of trials (but see Parker, 2019; Vasishth et al., 2008; Wagers, 2008, for some perspectives on this issue).

Still, consideration of the basic assumptions of both models suggests that in principle, both should predict that the ungrammatical attraction configuration tested here slows processing across the board, rather than only when the ungrammaticality is detected. A continuous number valuation account claims that the representation of the subject’s number is degraded when there is a mismatching attractor, and standard activation-based cue-based retrieval theories claim that the process of retrieving the agreement controller takes longer when no noun perfectly matches the verb’s retrieval cues; these conditions hold in the ungrammatical attraction configuration, regardless of how the sentence is ultimately judged.

Thus, if the present results indicate that there is simply no processing difficulty when a comprehender ‘falls for’ the illusion of grammaticality in the agreement attraction configuration, these results would seem problematic for both accounts of agreement attraction. However, the present results also admit of an interpretation according to which there is, in fact, difficulty in sentences like (5a) even when they are judged to be grammatical, but this difficulty simply does not disrupt the forward progress of the eyes. We wish to be perfectly explicit that we cannot, at present, arbitrate between these two interpretations. Either the agreement error causes no difficulty on those occasions on which the reader does not notice it—a conclusion that would seem very important for a theoretical understanding of agreement attraction—or it does cause some difficulty that does not impede the forward movement of the eyes; we do not know which.

We also note the possibility that when the reader does not explicitly notice the agreement violation in *The editor of the magazines are*, this is because she has rapidly and automatically made an inference that the subject was actually plural, not singular. Some previous studies (Brehm et al., 2021; Patson & Husband, 2016) have found that comprehenders do sometimes make this inference in this ungrammatical attraction configuration. However, it has also been argued that such ‘noisy channel’ inference (Gibson et al., 2013) would be expected to show a cost in the eye movement record (Cutter et al., 2024). Thus, the lack of cost is arguably no less surprising on this inference-based view of failure to notice agreement errors.

### Incremental Processing Disruption and Recognition of Grammatical Errors

It is striking that the explicit conscious recognition of a grammatical error is so tightly associated with immediate and pronounced disruption during free reading. We note that this tight relationship is evident not only in the experiments we report here, but in previous experiments using the IJP (Huang & Staub, 2021; Staub et al., 2024). One obvious question about this relationship, however, is about the nature of the temporal and causal connection. Does eye movement disruption result from, and temporally follow, initial conscious awareness of an error? Or, do both conscious awareness of an error and eye movement disruption result from the failure of unconscious syntactic parsing processes, such as a process of agreement checking, with no direct causal link between the two? We cannot answer this question at present. Nevertheless, the close relationship between incremental processing disruption and subsequent acceptability judgments could prove useful for understanding the cognitive processes that culminate in linguistic acceptability judgments. Our data support a view of conscious acceptability judgments as reflecting in part an error signal that spontaneously arises during the processing of linguistic stimuli (Barrios, 2012; Sprouse, 2008).

### Can ‘Integration Failure’ Account for All Syntactic Processing Effects on Eye Movements?

In the present study we have shown that eye movement effects in ungrammatical agreement attraction sentences can be understood as arising entirely from integration failure on some trials, with no eye movement effects appearing on other trials. We have also suggested above that garden path effects on eye movements plausibly arise from a similar mechanism. However, we acknowledge that there are other examples of syntactic effects on eye movements that may challenge this framework. For example, Bartek et al. (2011) observed that increasing subject-verb distance can slow down reading. It is not clear that such locality effects can be explained in terms of integration failure or error detection, nor that they can be reduced to surprisal. More broadly, locality and related principles governing memory retrieval have been shown to predict reading times above and beyond surprisal in several independent studies (Demberg & Keller, 2008; Isono, 2024; Ryu & Lewis, 2025). Thus, we do not, at present, commit to the complete absence of syntactic effects on eye movement in reading in the absence of integration failure; instead, we only offer evidence that at least some well-established syntactic effects can be understood in terms of an all-or-none integration failure mechanism.

## CONCLUSION

Two experiments investigated the relationship between explicit, conscious recognition of a subject-verb agreement error and incremental eye movement measures. The main conclusion of these experiments is very clear: An eye movement disruption associated with computing agreement is present when, and only when, the reader recognizes the error. Together with previous studies using a similar paradigm (e.g., Staub et al., 2024), the present study confirms the assumption made by the E-Z Reader 10 model (Reichle et al., 2009) that post-lexical processing affects eye movements in reading only when such post-lexical processing fails. While it remains possible that this conclusion does not hold for all types of post-lexical processing, the present data already call for reconsideration of the intuitive notion that syntactic processing difficulty is straightforwardly reflected in incremental reading time measures.

## ACKNOWLEDGMENTS

We thank the following research assistants for their invaluable assistance in data collection: Murrette Gaudette, Aarohee Gondkar, John Greene, Jillian Hammond, Izabella Jenkins, and Yakira Ramirez. We are grateful to the audiences at the UMass Cognition and Cognitive Neuroscience colloquium and the Potsdam University Sentence Processing Workshop for helpful feedback, and to Byung-Doh Oh, Grusha Prasad, Kuan-Jung Huang, Suhas Arehalli, Tal Linzen, and William Timkey for discussion.

## FUNDING INFORMATION

During the preparation of this manuscript B.D. was supported in part by a Samuel F. Conti fellowship from the University of Massachusetts, Amherst, and by NSF IIS 2504954 to the University of Massachusetts, Amherst.

## AUTHOR CONTRIBUTIONS

A.S.: Conceptualization; Data analysis; Data curation; Supervision; Writing – original draft. N.W.: Conceptualization; Data analysis; Writing – review & editing. B.D.: Conceptualization; Data analysis; Writing – original draft.

## GENERATIVE AI STATEMENT

Generative AI tools were used to generate experimental stimuli, typeset math formulae, and edit and review analysis code written by the authors.

## DATA AVAILABILITY STATEMENT

Materials, item statistics, eye movement data, and analysis scripts are available in the following OSF repository: https://osf.io/4xzf5/overview. The experiments were not pre-registered.

## Notes

^1^ Of course, it is possible in principle for an initial integration failure to be repaired, which would presumably result in the reader responding ‘no’ to the judgment question; for example, a reader may experience disruption in reading a garden path sentence, but then recover the correct syntactic analysis. However, the sentences that have been investigated using the IJP, and the ones we investigate in the present study, are fully ungrammatical, so we do not expect initial integration failure to be followed by successful repair.^2^ The initial ChatGPT prompt was as follows: “I’d like you to create sentences for an experiment on agreement attraction. I’d like them to start with a subject that is modified by a prepositional phrase, like ‘the key to the cabinets’, and have a form of the verb “to be” in the verb phrase. The sentence should continue for at least 3 or 4 words after the verb.” The second prompt was as follows: “Now make another set, but this time, put some additional material, such as an adverbial phrase, before the subject.”^3^ Gulordava et al.’s English language model is a two-layer LSTM model trained on 90 M tokens of English Wikipedia text. A limitation of the present analysis is that we use only a single language model, making it possible that the results would not generalize beyond this specific choice. However, we note that studies that have used a greater diversity of language models, like Wilcox et al. (2021) and Huang et al. (2024), have not observed large differences in the magnitude of RT predictions for targeted experimental contrasts across models.^4^ This modification of the van Schijndel and Linzen method responds to a request from a reviewer of a previous version of this manuscript. We note that the Bayesian formulation of the surprisal generalization analysis that we develop here yields results that are qualitatively similar to the results of an analysis that more directly implements van Schijndel and Linzen’s original methodology.^5^ Note that the credible interval on the AU-AG comparison in go-past times includes 0. In that sense this result (which is based on a model fit to raw RTs) diverges from the results reported in Table 3 (which are based on a model fit to log-transformed RTs). The credibility of the AU-AG effect in go-past times should be interpreted with this in mind.^6^ The *lmer* syntax for this model is *RT* ∼ *s*_0_ + *s*_−1_ + *s*_−2_ + *f*_0_ * *l*_0_ + *f*_−1_ * *l*_−1_ + *f*_−2_ * *l*_2_ + (1 | *item*) + (1 + *s*_0_ | *participant*).

## APPENDIX A: BAYESIAN SURPRISAL GENERALIZATION ANALYSIS

van Schijndel and Linzen (2021) proposed a method for generating reading time predictions from surprisal values of a language model. Their method involves training a linear regression model on a set of *training* or *filler* data to estimate the effect of surprisal on reading times for a given set of participants in a given experimental setting. The regression coefficients associated with the surprisal predictors are referred to as *conversion factors*. The conversion factors are the basis for generating predictions about reading time data in a set of held-out test data. These held-out test data typically come from factorial experimental contrasts, such as the difference between two experimental conditions in a test set of data. The result of this *surprisal generalization analysis* is a prediction of the effect size of such an experimental manipulation conditioned on the surprisal from a given language model alone.

Here we describe a method for implementing this core analysis in a fully Bayesian framework. We note that this analysis departs in several places from the original van Schijndel and Linzen (2021) analysis pipeline, but provides qualitatively similar results on our experimental data.

### Step 1: Estimate $\hat{P}$(*θ*_*f*_|***F***)

Let ***F*** = {*F*_1_, …, *F*_*P*_} be our filler data, where each *F*_*p*_ is a set of reading time measurements on a set of filler sentences for the *p*th out of *P* participants in our dataset. The reading time measurements consist in the different reading time measurements computed over the fixation record in any given trial: first pass, go-past, and total reading times.

We follow Huang et al. (2024) and van Schijndel and Linzen (2021) in fitting a linear mixed effects regression model to the reading times in ***F***, with predictor variables that include surprisal from a given language model, as well as lexical control variables for the current word and two previous words.

The full filler model is:

$$\begin{array}{l} RT_{ip}=\alpha +u_{p}+\gamma_{j\left(i\right)}+\left(\beta_{s_{0}}+b_{p}\right)s_{i,0}+\beta_{s_{-1}}\;s_{i,-1}+\beta_{s_{-2}}\;s_{i,-2}\\ \;+\beta_{f_{0}}\;f_{i,0}+\beta_{l_{0}}\;l_{i,0}+\beta_{f_{0}l_{0}}\;f_{i,0}\;l_{i,0}\\ \;+\beta_{f_{-1}}\;f_{i,-1}+\beta_{l_{-1}}\;l_{i,-1}+\beta_{f_{-1}l_{-1}}\;f_{i,-1}\;l_{i,-1}\\ \;+\beta_{f_{-2}}\;f_{i,-2}+\beta_{l_{-2}}\;l_{i,-2}+\beta_{f_{-2}l_{-2}}\;f_{i,-2}\;l_{i,-2}+\epsilon_{ip} \end{array}$$

where *i* indexes words, *p* indexes participants, and *j*(*i*) indexes the item (sentence) containing word *i*. The predictors *s*_*i*,*k*_, *f*_*i*,*k*_, and *l*_*i*,*k*_ denote the surprisal, (centered) log frequency, and (centered) length of the word at position *k* ∈ {0, −1, −2} relative to word *i*, respectively. The random-effects structure is:

$$\left(\begin{array}{c} u_{p}\\ b_{p} \end{array}\right)∼\text{Normal}\left(0,\Sigma_{\text{part}}\right),\;\gamma_{j}∼\text{Normal}\left(0,\sigma_{\text{item}}^{2}\right),\;\epsilon_{ip}∼\text{Normal}\left(0,\sigma^{2}\right)$$

where *u*_*p*_ and *b*_*p*_ are by-participant random intercepts and slopes on *s*_*i*,0_ (only), and *γ*_*j*_ is a by-item random intercept.^6^

The full parameter vector of interest for our filler model, *θ*_*f*_, is therefore:

$$\begin{array}{l} \theta_{f}=\{\alpha ,\beta_{s_{0}},\beta_{s_{-1}},\beta_{s_{-2}},\beta_{f_{0}},\beta_{l_{0}},\beta_{f_{0}l_{0}},\beta_{f_{-1}},\beta_{l_{-1}},\beta_{f_{-1}l_{-1}},\beta_{f_{-2}},\beta_{l_{-2}},\beta_{f_{-2}l_{-2}},\\ \;u_{1},\ldots ,u_{p},b_{1},\ldots ,b_{p},\gamma_{1},\ldots ,\gamma_{j},\sigma^{2},\sigma_{\text{item}}^{2},\Sigma_{\text{part}}\} \end{array}$$

We estimate $\hat{P}$(*θ*_*f*_|***F***) by fitting the filler model to our filler data using *brms*. We used raw reading times as our dependent variable and ran four independent chains for 4000 samples each. All Rhats were 1.0, and no divergences were observed. The prior specifications are detailed below.

The posterior distribution $\hat{P}$(*θ*_*f*_|***F***) is thus represented as a set of *M* draws:

$${\left\{\theta_{f}^{\left(m\right)}\right\}}_{m=1}^{M}={\left\{\alpha^{\left(m\right)},\beta_{s_{0}}^{\left(m\right)},\ldots ,\beta_{f_{-2}l_{-2}}^{\left(m\right)},u_{1}^{\left(m\right)},\ldots ,u_{p}^{\left(m\right)},b_{1}^{\left(m\right)},\ldots ,b_{p}^{\left(m\right)},\gamma_{1}^{\left(m\right)},\ldots ,\gamma_{j}^{\left(m\right)},\sigma^{\left(m\right)},\Sigma_{\text{item}}^{\left(m\right)},\Sigma_{\text{part}}^{\left(m\right)}\right\}}_{m=1}^{M}$$

These draws represent uncertainty in the conversion factor coefficients {*β*_*s*_0__, *β*_*s*_−1__, *β*_*s*_−2__}, along with any covariation in those draws with other parameters that will be important for generating predicted RTs, such as the by-subject intercepts and slopes.

### Step 2: Compute the Predictive Distribution on the Predicted Agreement Cost, $\hat{P}$(*A*^pred^|***F***, ***Q***)

We wish to predict the contrast between the reading time on a critical verb in two conditions in our experiment, one with grammatical agreement features and one with ungrammatical agreement features. We do this by computing the predictive distribution on this cost, given $\hat{P}$(*θ*_*f*_|***F***). Specifically, we take $\hat{P}$(*θ*_*f*_|***F***) as estimated in Step 1, and compute the predictive distribution on a new latent variable: the *predicted agreement cost*, *A*^pred^. This latent variable represents the predicted RT difference between the two relevant conditions in our experiment; these are labeled conditions 0 and 1 here.

For a given participant *p* and a given experimental item *q* (out of *Q* total experimental items), appearing in conditions 0 (grammatical) and 1 (ungrammatical), the model-predicted RT in each condition can be computed as:

$$\begin{array}{l} {\hat{RT}}_{qp0}=\left(\alpha +u_{p}+\gamma_{q}\right)+\left(\beta_{s_{0}}+b_{p}\right)S_{q,0}^{0}+\beta_{s_{-1}}\;S_{q,-1}^{0}+\beta_{s_{-2}}\;S_{q,-2}^{0}\\ \;+\beta_{f_{0}}\;f_{q,0}+\beta_{l_{0}}\;l_{q,0}+\beta_{f_{0}l_{0}}\;f_{q,0}\;l_{q,0}\\ \;+\beta_{f_{-1}}\;f_{q,-1}+\beta_{l_{-1}}\;l_{q,-1}+\beta_{f_{-1}l_{-1}}\;f_{q,-1}\;l_{q,-1}\\ \;+\beta_{f_{-2}}\;f_{q,-2}+\beta_{l_{-2}}\;l_{q,-2}+\beta_{f_{-2}l_{-2}}\;f_{q,-2}\;l_{q,-2}\\ {\hat{RT}}_{qp1}=\left(\alpha +u_{p}+\gamma_{q}\right)+\left(\beta_{s_{0}}+b_{p}\right)S_{q,0}^{1}+\beta_{s_{-1}}\;S_{q,-1}^{1}+\beta_{s_{-2}}\;S_{q,-2}^{1}\\ \;+\beta_{f_{0}}\;f_{q,0}+\beta_{l_{0}}\;l_{q,0}+\beta_{f_{0}l_{0}}\;f_{q,0}\;l_{q,0}\\ \;+\beta_{f_{-1}}\;f_{q,-1}+\beta_{l_{-1}}\;l_{q,-1}+\beta_{f_{-1}l_{-1}}\;f_{q,-1}\;l_{q,-1}\\ \;+\beta_{f_{-2}}\;f_{q,-2}+\beta_{l_{-2}}\;l_{q,-2}+\beta_{f_{-2}l_{-2}}\;f_{q,-2}\;l_{q,-2} \end{array}$$

where $S_{q,k}^{0}$ and $S_{q,k}^{1}$ are the surprisal values at position *k* relative to the critical word of item *q* in conditions 0 and 1, respectively, and *f*_*q*,*k*_ and *l*_*q*,*k*_ are the log frequency and length of the word at position *k* relative to the critical word of item *q*.

The predicted agreement cost for participant *p* on item *q* is their difference: $A_{qp}^{\text{pred}}={\hat{RT}}_{qp1}-{\hat{RT}}_{qp0}$. We may reduce this expression considerably, given that many terms in each predicted RT do not vary with condition. For example, the intercepts *α*, *u*_*p*_, and *γ*_*q*_ all cancel. In addition, the frequency and length terms at all positions will also cancel, by virtue of our experimental design: the critical word and the two preceding words are identical across conditions 0 and 1. This leaves only coefficients associated with surprisal, which may differ by condition at all word positions considered in the model. This is because surprisal is assigned by a language model conditioned on the full preceding context, and that the two conditions do differ in their distal context. For notational convenience, we can define the difference in surprisal on a given word between our two experimental conditions within a given item:

$$\Delta S_{q,k}\equiv S_{q,k}^{1}-S_{q,k}^{0},\;k\in \left\{0,-1,-2\right\}$$

With this, we may reduce the calculation of the predicted agreement cost to:

$$A_{qp}^{\text{pred}}={\hat{RT}}_{qp1}-{\hat{RT}}_{qp0}=\left(\beta_{s_{0}}+b_{p}\right)\Delta S_{q,0}+\beta_{s_{-1}}\;\Delta S_{q,-1}+\beta_{s_{-2}}\;\Delta S_{q,-2}$$

Note that the participant random slope *b*_*p*_ applies only to the position 0 surprisal term, consistent with the random-effects structure of the filler model defined above. Note that this reduction therefore reflects the specific random effects structure of the filler model above, and would need to be modified if a different parameterization of the filler model is adopted.

The predicted agreement cost above represents the predicted cost associated with two conditions in a single experimental itemset *q* in our experiment. To get the full predicted agreement cost in our experiment, we average across participants and items:

$$A^{\text{pred}}=g\left(\theta_{f}\right)=\frac{1}{QP}\sum_{p=1}^{p}\sum_{q=1}^{Q}\left[\left(\beta_{s_{0}}+b_{p}\right)\Delta S_{q,0}+\beta_{s_{-1}}\;\Delta S_{q,-1}+\beta_{s_{-2}}\;\Delta S_{q,-2}\right]$$

Monte Carlo draws of the predicted agreement cost given our filler items and experimental stimuli, $\hat{P}$(*A*^pred^|***F***, ***Q***), can be computed from the Monte Carlo draws of $\hat{P}$(*θ*_*f*_|***F***), allowing for principled propagation of uncertainty in the filler model through to our estimate of *A*^pred^.

$$A^{\text{pred},\left(m\right)}=\frac{1}{QP}\sum_{p=1}^{p}\sum_{q=1}^{Q}\left[\left(\beta_{s_{0}}^{\left(m\right)}+b_{p}^{\left(m\right)}\right)\Delta S_{q,0}+\beta_{s_{-1}}^{\left(m\right)}\;\Delta S_{q,-1}+\beta_{s_{-2}}^{\left(m\right)}\;\Delta S_{q,-2}\right]$$

The predicted agreement cost can be extended to a two-word region spanning the critical auxiliary and the immediately following participial. The aggregated predicted difficulty is simply the sum of the predicted agreement costs at the two positions:

$$A_{\text{region},qp}^{\text{pred}}=A_{aux,qp}^{\text{pred}}+A_{\text{part},qp}^{\text{pred}}$$

where $A_{aux,qp}^{\text{pred}}$ and $A_{\text{part},qp}^{\text{pred}}$ are computed from the filler model applied to the auxiliary and participial words in the critical region, respectively. The aggregated agreement cost has the following Monte Carlo draws:

$$A_{\text{region}}^{\text{pred}}=\frac{1}{QP}\sum_{p=1}^{p}\sum_{q=1}^{Q}\left(A_{aux,qp}^{\text{pred}}+A_{\text{part},qp}^{\text{pred}}\right)$$

$$A_{\text{region}}^{\text{pred},\left(m\right)}=\frac{1}{QP}\sum_{p=1}^{p}\sum_{q=1}^{Q}\left(A_{aux,qp}^{\text{pred}}+A_{\text{part},qp}^{\text{pred}}\right)$$

In models fit in the surprisal generalization analysis, we place the following priors on the parameters of models (recall that both the filler and empirical models are fit on raw, not log-transformed RTs). For the fixed effects:

$$\alpha ∼\text{Normal}\left(500,200\right),\;\beta_{\cdot }∼\text{Normal}\left(0,100\right)$$

where *α* is the intercept, and *β*_·_ denotes any fixed-effect coefficient.

For the variance components, the covariance matrix of the by-participant random effects is:

$$\Sigma_{\text{part}}∼{\text{Normal}}^{+}\left(0,100\right)\times \text{LKJ}{\left(2\right)}_{cor}$$

The item-level variance and residual standard deviations are:

$$\sigma_{\text{item}}∼{\text{Normal}}^{+}\left(0,100\right),\;\sigma ∼{\text{Normal}}^{+}\left(0,100\right)$$

where Normal^+^ (0, 1) denotes a half-normal distribution.
